# Mitochondria-associated membrane protein PACS2 maintains right cardiac function in hypobaric hypoxia

**DOI:** 10.1016/j.isci.2023.106328

**Published:** 2023-03-05

**Authors:** Jie Yang, Mengjia Sun, Renzheng Chen, Xiaowei Ye, Boji Wu, Zhen Liu, Jihang Zhang, Xubin Gao, Ran Cheng, Chunyan He, Jingyu He, Xuhong Wang, Lan Huang

**Affiliations:** 1 Institute of Cardiovascular Diseases of PLA, the Second Affiliated Hospital, Army Medical University (Third Military Medical University), Chongqing, China; 2 Department of Cardiology, the Second Affiliated Hospital, Army Medical University (Third Military Medical University), Chongqing, China

**Keywords:** Cardiovascular medicine, Physiology, Molecular biology, Proteomics, Metabolomics

## Abstract

Hypobaric hypoxia (HH) is the primary challenge at highland. Prolonged HH exposure impairs right cardiac function. Mitochondria-associated membrane (MAM) plays a principal role in regulating mitochondrial function under hypoxia, but the mechanism was unclear. In this study, proteomics analysis identified that PACS2, a key protein in MAM, and mitophagy were downregulated in HH. Metabolomics analysis indicated suppression of glucose and fatty acids aerobic oxidation in HH conditions. Cardiomyocyte *Pacs2* deficiency disrupted MAM formation and endoplasmic reticulum (ER)-mitochondria calcium flux, further inhibiting mitophagy and energy metabolism in HH. *Pacs2* overexpression reversed these effects. Cardiac-specific knockout of *Pacs2* exacerbated mitophagy inhibition, cardiomyocyte injury, and right cardiac dysfunction induced by HH. Conditional knock-in of *Pacs2* recovered HH-induced right cardiac impairment. Thus, PACS2 is essential for protecting cardiomyocytes through ER-mitochondria calcium flux, mitophagy, and mitochondrial energy metabolism. Our work provides insight into the mechanism of HH-induced cardiomyocyte injury and potential targets for maintaining the right cardiac function at the highland.

## Highlights

• PACS2 is essential for cardiac function maintenance at highland
• Hypobaric hypoxia decreases PACS2, mitophagy and energy metabolism
• PACS2-mediated MAM calcium flux is required for mitophagy and energy metabolism
• PACS2 supplement restores mitophagy, energy metabolism, and right cardiac function

## Graphical abstract

**Figure:**
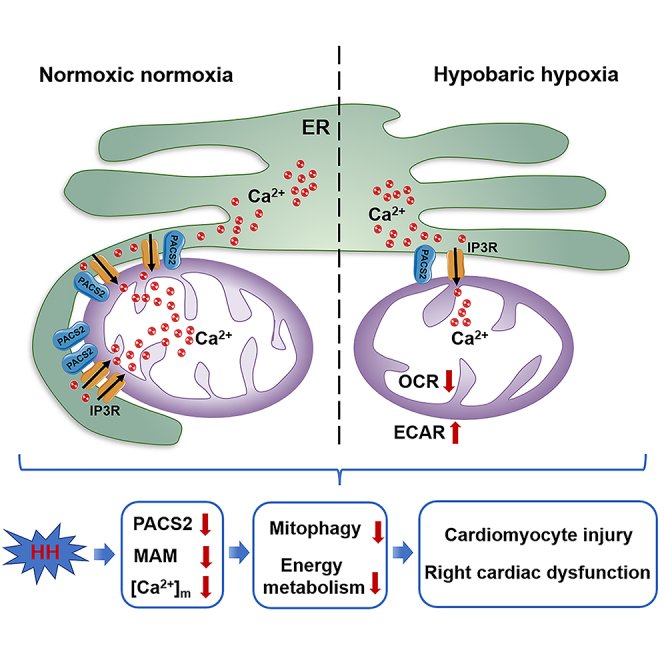

## Introduction

High-altitude areas cover much of the total geographical area worldwide, and human exposure to high altitudes is increasing for various reasons. Hypobaric hypoxia (HH) caused by exposure to increasing altitude is the main physiological challenge in such conditions and has long been recognized as a cause of cardiac stress. Acute exposure to high altitudes induces an increase in the right ventricular (RV) afterload, leading to the alteration of the RV filling patterns.^1^ Prolonged exposure to high altitude further results in chronic remodeling of the cardiac structure and function, ultimately leading to right heart failure.^2^^,^^3^ Among these cardiac adaptive and/or pathological alterations, cardiomyocyte responses, particularly in intracellular homeostasis maintenance during hypoxia, are the critical molecular basis that determines the adaptive cardiac outcomes.^4^^,^^5^

Cardiomyocytes consume the majority of oxygen in the mitochondria as an electron donor for oxidative phosphorylation (OXPHOS).^6^^,^^7^^,^^8^ Thus, mitochondria are highly sensitive to decreases in oxygen levels in cardiomyocytes. Hypoxia increases oxidative stress and mitochondrial DNA mutation and causes mitochondrial dysfunction.^9^ Hypoxia also suppresses mitochondrial OXPHOS and leads to the accumulation of aerobic metabolic substrates and anaerobic metabolites, which result in cardiomyocyte injury and cardiac dysfunction.^10^ These damaged mitochondria and excessive metabolic substrates could be removed via mitophagy.^11^^,^^12^ However, mitophagy is suppressed in some cardiac diseases, such as diabetic cardiomyopathy and ischemic cardiomyopathy,^13^ which leads to mitochondrial dyshomeostasis and cardiac dysfunction. Therefore, an appropriate level of mitophagy serves as a protective mechanism to maintain the mitochondrial function in response to cardiac stress.^14^ In recent years, several mitochondrial membrane receptors containing the LC3-interacting region (LIR) motif have been found to mediate mitophagosome formation under acute hypoxic conditions.^11^^,^^15^ However, there is a paucity of information regarding cardiomyocyte mitophagy during chronic HH exposure, and the precise mechanism has not been fully elucidated.

Recent studies indicated that cardiomyocyte mitochondrial function is regulated by the mitochondria-associated membrane (MAM) structure, tethering two organelles by proteins located on opposing membranes.^16^^,^^17^^,^^18^ Stable contact between the ER and the mitochondria integrates the two organelles’ functions. Dysfunctional communications between organelles are implicated in cardiovascular disorders, neurodegeneration,^19^ diabetes^20^ and cancer.^21^ Phosphofurin acidic cluster sorting protein 2 (PACS2) is a crucial physical linkage protein on the MAM, connecting the ER with the mitochondria and maintaining MAM integrity and function. It plays important roles in many cellular activities, such as lipid synthesis, calcium signaling, autophagy, and apoptosis. Simmen et al. first found that PACS2 depletion causes extensive mitochondrial fragmentation, and fragmented mitochondria uncouple from the ER.^22^ Subsequently, researchers have also demonstrated that PACS2 depletion induced severe cardiovascular diseases.^19^ For example, downregulation of PACS2 by miRNA-182 remarkably inhibited cardiomyocyte apoptosis in the progress of heart failure.^23^ In diabetic cardiomyopathy, hyperglycaemia caused distortion of MAM formation via PACS2, IP3R2, FUNDC1, and VDAC1 and decreased mitochondrial biogenesis, fusion and OXPHOS, which contributes significantly to fibrosis and hypertrophy in the heart.^24^ Meanwhile, PACS2 also regulates autophagic and mitophagic flux. The knockdown of PACS2 results in a reduction in the autophagosome marker LC3 II in starved cells, which is associated with the inhibition of STX17-dependent ATG14-recruitment to MAM sites.^25^ Removal of PACS2 interrupts mitophagosome formation in MAMs, which subsequently impairs mitophagy and involves the process of cell apoptosis in response to stress.^26^ Notably, the re-expression of PACS2 could reverse these changes. As a protein with the potential function of autophagic and metabolic regulation, whether PACS2 changes in MAM are related to cardiomyocyte mitophagy and metabolism under HH condition is still worth studying.

In this study, we established HH conditions to closely simulate high-altitude exposure and focused on PACS2-mediated mitophagy and mitochondrial energy metabolism, regarding calcium flux across the MAM as the core mechanism. This study provides insights into the cardiomyocyte response to HH conditions. We also interpreted the mechanism underlying high-altitude-induced right cardiac dysfunction.

## Results

### Hypobaric hypoxia exposure induces the downregulation of PACS2 and affects mitophagy and mitochondrial energy metabolism in the right myocardium

**Figure 1:**
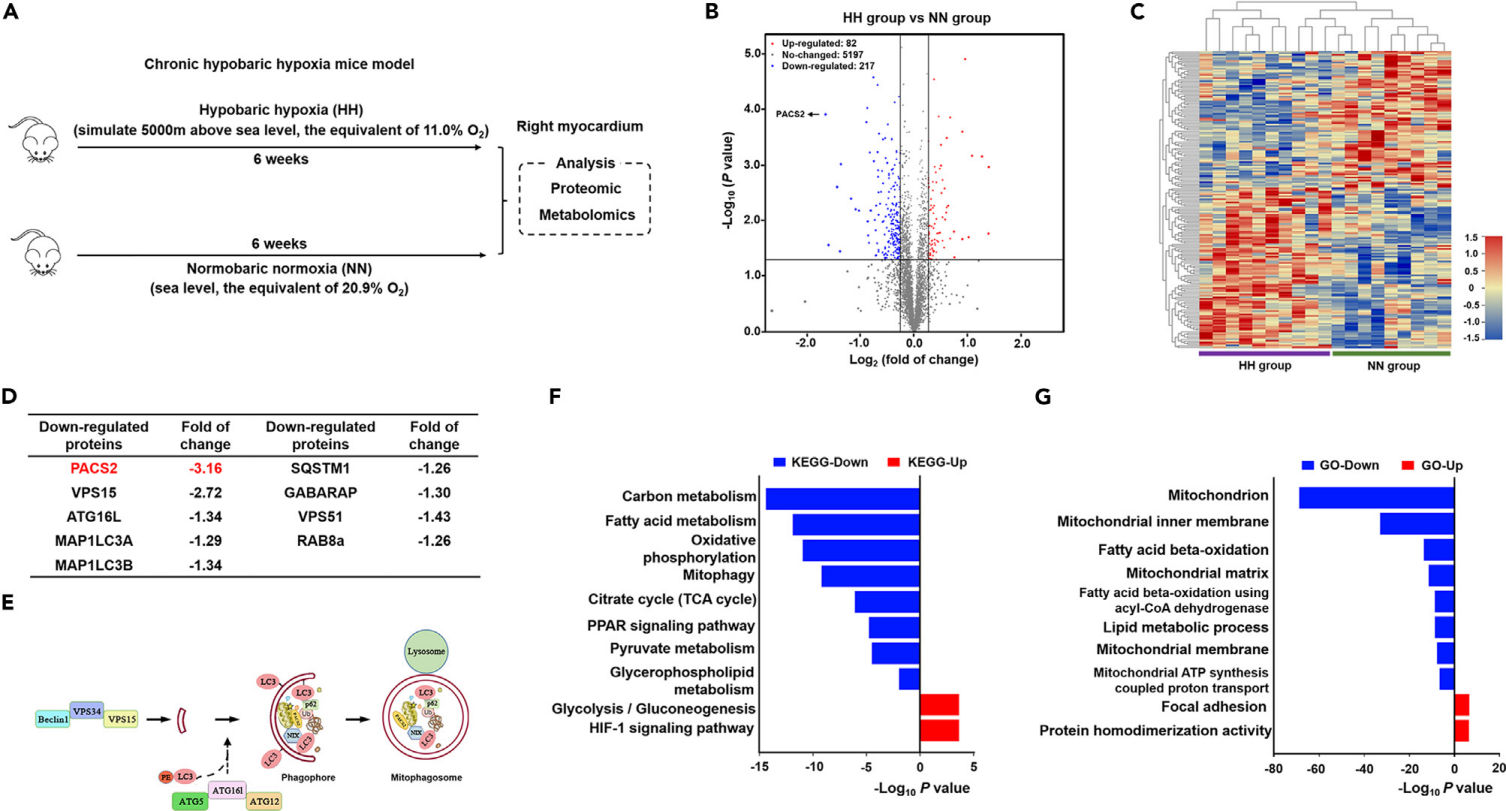
Proteomics and metabolomics assessment of the right myocardium in response to hypobaric hypoxia (A) Protocol for establishing chronic HH-induced mice model and related omics analysis. (B) Volcano plot indicating the number of significantly upregulated (red, n = 82) and downregulated (blue, n = 217) proteins via proteomics analysis. Significantly downregulated PACS2 is indicated by arrows (fold of change = −3.16). (C) Sample cluster heatmap performed with hierarchical clustering in HH mice heart vs. NN controls are assessed by metabolomics analysis (adjusted p value <0.05). Red: up-regulation and blue: downregulation. The purple and green labels at the bottom of the heatmap represent the separation between the HH and NN groups. (D) Downregulation of PACS2 (fold change = −3.16) and mitophagy-related proteins in the HH group compared with NN controls. (E) Mechanism diagram of phagophore and mitophagosome formation with the participation of PACS2 and mitophagy-related proteins. (F and G) Bar graphs of the significantly upregulated (red) and downregulated (blue) terms by KEGG (F) and GO (G) pathways analysis. HH, hypobaric hypoxia; NN, normobaric normoxia; KEGG, Kyoto Encyclopedia of Genes and Genomes; GO, gene ontology.

C57BL/6J mice were assigned to an HH chamber for 6 weeks for the simulation of high-altitude conditions (Figure 1A). We first performed proteomics and metabolomics analyses accordingly in the right myocardium of mice that had been allowed to develop hypoxia-induced pulmonary hypertension after the 6-week chronic HH exposure. The different candidates were defined using a criterion of ≥1.2 log_2_ fold change and a significant difference between the groups. In the proteomics analysis, we identified 217 downregulated proteins and 82 upregulated proteins in the right myocardium of HH-exposed mice when compared with the respective levels in the normobaric normoxia (NN) counterparts (Figure 1B and Table S1). In the metabolomics analysis, we identified that 53 endogenous metabolites increased and 22 decreased (Table S2). Hierarchical clustering analysis of metabolomics indicated markedly altered cardiac metabolic pathways under HH exposure (Figure 1C). Among the differentially expressed proteins, we identified PACS2 as being significantly downregulated by 3.16-fold (Figure 1D). In addition to PACS2, MAP1LC3A, MAP1LC3B, autophagy-related 16-like 1, and sequestosome 1 (SQSTM1/p62) were remarkably downregulated; these were involved in phagophore formation and mitophagy induction (Figure 1E). To gain insight into the possible biological effect of HH exposure, we subjected proteins that were down- or upregulated to the Kyoto Encyclopedia of Genes and Genomes (KEGG) and Gene Ontology (GO) pathway enrichment analysis. Glycolysis, the HIF-1 signaling pathway, and focal adhesion were upregulated during HH, while OXPHOS, the citrate cycle, fatty acid beta-oxidation, and mitophagy were downregulated (Figures 1F and 1G). The above-mentioned results indicated that both mitophagy and mitochondrial energy metabolism were impaired in the right myocardium of the mice owing to HH exposure.

### Cardiac *Pacs2* ablation exacerbated the right cardiac dysfunction and structure impairment induced via hypobaric hypoxia exposure

**Figure 2:**
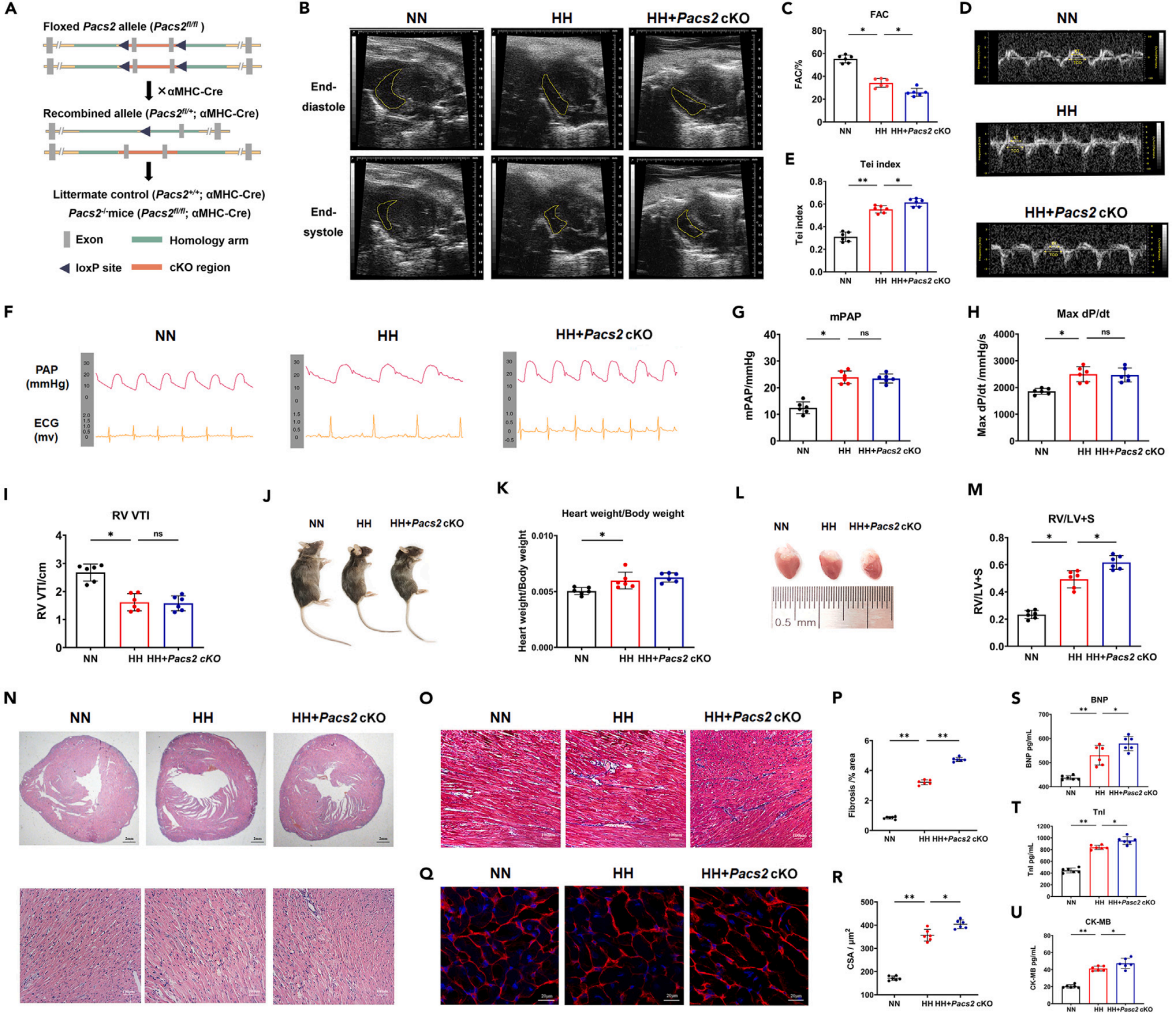
Cardiac *Pacs2* ablation exacerbated the right cardiac dysfunction and right myocardium structure impairment after hypobaric hypoxia (A) Schematic of cardiomyocyte-specific *Pacs2* cKO model. (B and C) FAC measurement of the RV in the NN group (FAC = 57.60%), HH group (FAC = 23.36%), and HH + *Pacs2* cKO group (FAC = 18.47%). Statistics data show mean ± standard error of mean (SEM) in (B). Representative images were acquired at end-diastole (up) and end-systole (down) (N = 6 hearts per group). (D and E) Tei index is measured in the NN (Tei index = 0.35), HH (Tei index = 0.50), and HH + *Pacs2* cKO groups (Tei index = 0.68) by tissue Doppler imaging. Statistics data show mean ± SEM in (E) (N = 6 hearts per group). (F and G) mPAP was measured by RHC in the NN (mPAP = 13.27 mmHg), HH (mPAP = 27.66 mmHg), and HH + *Pacs2* cKO groups (mPAP = 24.67 mmHg). Statistics data show mean ± SEM in (G) (N = 6 hearts per group). (H) Statistics of max dP/dt in the three groups are calculated respectively (N = 6 hearts per group). (I) Statistics of RV VTI in the three groups are calculated respectively (N = 6 hearts per group). (J) Gross appearance of the whole body of the NN, HH, and HH + *Pacs2* cKO groups. (K) Bar graphs showing the heart weight to body weight ratio of the three groups. (L) Gross appearance of the whole heart of the NN, HH, and HH + *Pacs2* cKO groups (N = 6 hearts per group). (M) Bar graphs showing Fulton’s index of the three groups. (N) Representative HE staining images of RV myocardium in the NN group, HH group, and HH + *Pacs2* cKO group (N = 6 hearts per group). Scale bar: 100 μm. (O) Representative Masson’s trichrome staining images of RV myocardium in NN, HH, and HH + *Pacs2* cKO groups. Scale bar: 100 μm. (P) Quantification revealing myocardial fibrosis area (blue) in the three groups. (Q) Representative images of RV tissue stained with WGA (red) to delineate sarcolemma and DAPI (blue) (N = 6 hearts per group). Scale bar: 20 μm. (R) Quantification of relative cardiomyocyte CSA in the three groups (N = 6 hearts per group). (S-U) Statistics of plasma concentrations of BNP, TnI, and CK-MB of the mice in the three groups. Cardiac function indexes are obtained from six mice per group. Data was shown by mean ± SD, ∗p < 0.05, ∗∗p < 0.01. BNP: brain natriuretic peptide; CK-MB: creatine kinase MB; CSA: cross-sectional area; DAPI: FAC: fractional area change; HE: hematoxylin-eosin; HH: hypobaric hypoxia; max dP/dt: maximum positive time derivative of left ventricular pressure; mPAP: mean pulmonary artery pressure; NN: normobaric normoxia; RHC: right heart catheterization; RV: right ventricular; TnI: troponin I; VTI: velocity time integral; WGA: wheat germ agglutinin.

To determine the role of PACS2 in maintaining right cardiac function and structure, we generated cardiomyocyte-specific *Pacs2* cKO (*Pacs2*^*flox/flox*^*/Cre*^*αMHC+/−*^) mouse models (Figure 2A). The *Pac*s2 cKO mice and their littermate controls (*Pacs2*^*+/+*^*/Cre*^*αMHC+/−*^) underwent echocardiography and right heart catheterization (RHC) evaluation after a 6-week HH exposure. Mice subjected to HH conditions showed a markedly lower RV fractional area change (FAC) (Figures 2B and 2C), concurrent with an increase in the Tei index (Figures 2D and 2E), mPAP (Figures 2F and 2G), and max dP/dt (Figure 2H) and lower velocity time integral (VTI) (Figure 2I), indicating impaired right cardiac function. During exposure to HH, *Pacs2* cKO mice exhibited a significantly lower FAC (Figures 2B and 2C) and higher Tei index (Figures 2D and 2E) than their littermate controls. Interestingly, we observed that other parameters reflecting the afterload, including the mPAP, max dP/dt, and RV VTI, were not further aggravated in *Pacs2* cKO mice during HH exposure (Figures 2G–2I). These data suggest that *Pacs2* ablation exacerbates right cardiac impairment partly independent of the RV afterload during HH exposure. Additionally, hypobaric hypoxic mice showed increased heart mass (Figures 2J and 2K) and Fulton’s index (Figures 2L and 2M); however, the heart weight was not further changed in *Pacs2* cKO mice (Figure 2K). hematoxylin-eosin (HE) staining further revealed increased RV chamber thickness, decreased RV chamber size, and disordered arrangement of RV myocardium caused by cardiac *Pacs2* ablation (Figure 2N). Masson’s trichrome staining showed significant collagen deposition in the right myocardial interstitial space after HH exposure (Figures 2O and 2P). The mean cross-sectional area (CSA) of the RV cardiomyocytes in the HH group was significantly larger than that in the NN group (Figures 2Q and 2R). Such substantial cardiac remodeling caused by HH was significantly more serious in the hearts of *Pacs2* cKO mice. The cardiomyocyte injury was also evidenced by plasma markers (B-type natriuretic peptide [BNP], TnI, and CK-MB), which were much higher in *Pacs2* cKO mice than in the remaining two groups (Figures 2S–2U). Our results indicated that HH-induced right cardiac impairment phenotype became more noticeable following *Pacs2* ablation.

To verify the separate role of *Pacs2* ablation, we evaluated the right cardiac function under NN conditions. Compared with littermate controls, *Pacs2* cKO mice showed a normal mPAP (Figures S1C and S1F), which was accompanied by impaired right cardiac function, as revealed by the lower RV FAC (Figures S1A and S1D) and an increased Tei index (Figures S1B and S1E). *Pacs2* cKO mice in NN exhibited significantly increased myocardial disorder and fibrosis (Figures S2A–S2C). Additionally, cardiomyocyte injury was also evident in NN conditions in the presence of *Pacs2* cKO (Figure S2D–S2H). In general, *Pacs2* ablation exacerbated the cardiomyocyte injury and right cardiac dysfunction; however, it did not act on the RV afterload during HH exposure.

### Cardiac *Pacs2* ablation exacerbated mitochondria-associated membrane disruption and mitophagy reduction induced via hypobaric hypoxia

**Figure 3:**
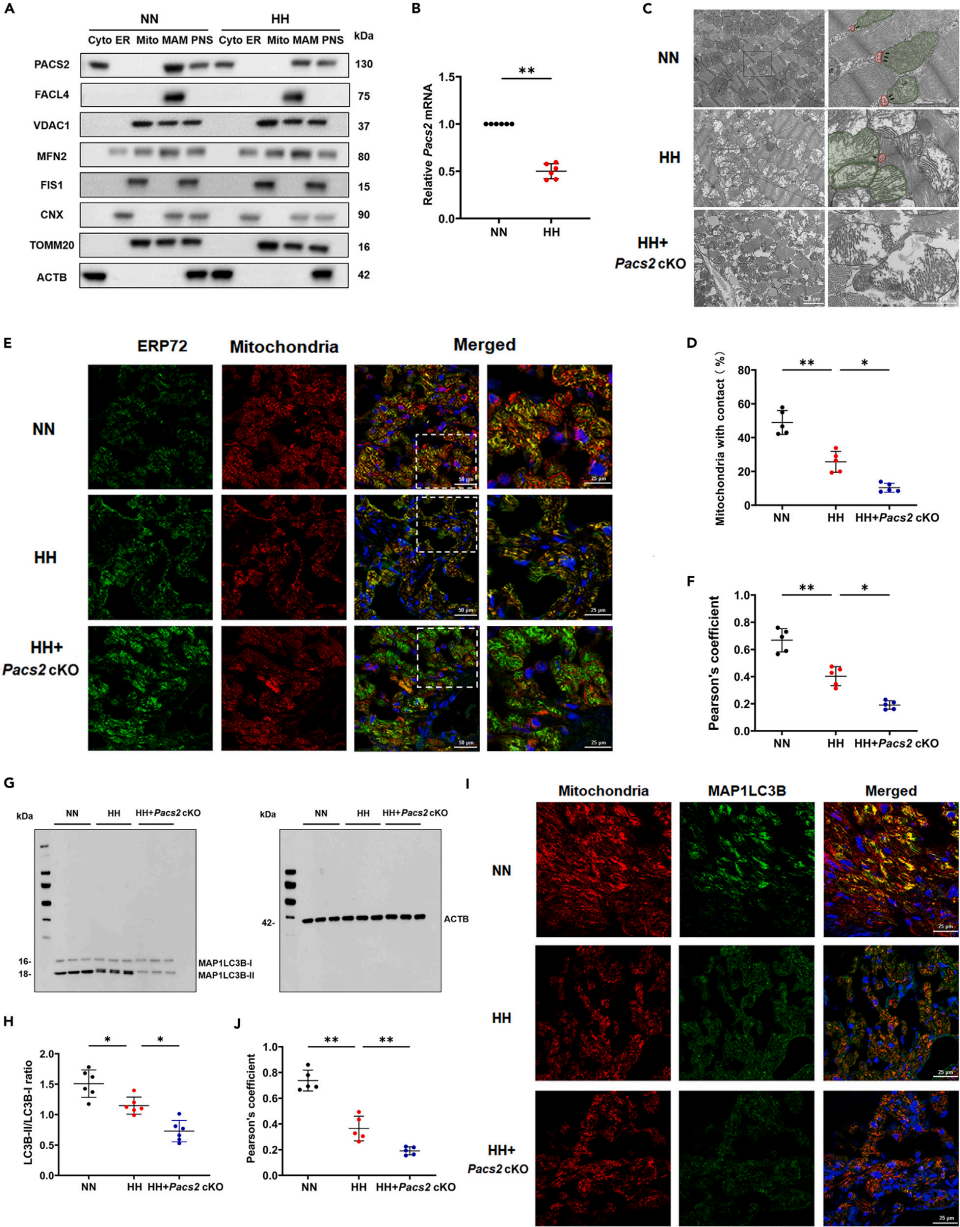
Cardiac *Pacs2* ablation exacerbated MAM disruption and mitophagy reduction induced by hypobaric hypoxia (A) Western blot analysis of PACS2 and MAM-related protein (FACL4, VDAC1, MFN2, FIS1, CNX, and TOMM20) levels in NN and HH conditions. (B) *Pacs2* gene expression in right myocardium in NN and HH conditions (N = 6 hearts per group). (C) Representative TEM images of MAM structure in NN, HH, and HH + *Pacs2* cKO groups (Scale bar: 2 μm) and then analyzed at higher magnification (Scale bar = 1 μm). ER are marked by red and mitochondria are marked by green. The black arrowheads indicate the ER-mitochondria contacts (<30 nm). (D) Quantification of ER and mitochondria contact in right myocardium (N = 6 hearts per group). Approximately 10–20 random fields with 50–100 mitochondria were analyzed in each experimental group. (E) Mitotracker Deep Red is used to mark mitochondria and ERP72 (green) is employed to mark ER in the right myocardium (Scale bar: 50 μm) and then analyzed at higher magnification (Scale bar: 25 μm). (F) Pearson’s overlap coefficient analysis indicates the lesser co-localization of ER with mitochondria (N = 6 hearts per group). (G and H) Representative western blots and statistical analysis (N = 6 hearts per group) of MAP1LC3B-I and MAP1LC3B-II in the three groups. (I) LSCM images of the right myocardium sections which were marked with Mitotracker Deep Red and MAP1LC3B (green). Scale bar: 25 μm. (J) Pearson’s overlap coefficient analysis of the co-localization of MAP1LC3B and mitochondria (N = 6 hearts per group). Data are shown as mean ± SD, ∗p < 0.05, ∗∗p < 0.01. CNX: calnexin; CYTO: cytosol; ER: ER FACL: fatty acid CoA ligase 4; FIS1: mitochondrial fission 1; HH: hypobaric hypoxia; LSCM: laser scanning confocal microscopy; MAM: mitochondria-associated membranes; MFN2: mitofusin 2; MITO: mitochondria; NN: normobaric normoxia; PNS: post-nuclear supernatant; TEM: transmission electron microscopy; TOMM20: translocase of the outer mitochondrial membrane member 20; VDAC1: voltage-dependent anion channel 1.

To determine how PACS2 responds to HH, we compared the subcellular localization of MAM-associated proteins in isolated right myocardium under HH or NN conditions. As shown in Figure 3A, different fractions were identified with the following organelle markers: FACL4, VDAC1, MFN2, FIS1, CNX, and TOMM20. The level of PACS2 in MAM significantly decreased in the HH group compared with its levels in the NN counterparts, although a small amount of PACS2 could also be found in the cytosol. However, the levels of other MAM-related proteins were not noticeably altered in MAM fractions isolated from the HH group. Notably, the *Pacs2* gene expression was also significantly reduced in HH hearts when compared with NN controls (Figure 3B). To assess whether PACS2 affects MAM integrity, we examined the ER-mitochondrial contacts in *Pacs2* cKO mice myocardium. As illustrated by the TEM images (Figures 3C and 3D), the proportion of ER in close contact with mitochondria relative to the total ER content was lower in the HH group than in the NN group and further decreased in the *Pacs2* cKO mice. Consistent with the TEM images, immunofluorescence analysis clearly showed a lower level of co-localization of the ER with mitochondria in the *Pacs2* cKO mice than in the remaining groups (Figures 3E and 3F).

Further, we determined whether PACS2 alteration affected mitophagy. Decreased mitophagy markers confirmed impaired mitophagy induced by HH exposure in the right myocardium. The blotting results indicated that the level of MAP1LC3B-II was lower in the *Pacs2* cKO mice than in controls during HH conditions (Figures 3G and 3H). Moreover, immunostaining analysis revealed that *Pacs2* deletion further reduced the co-localization of MAP1LC3B puncta and mitochondria induced by HH exposure (Figures 3I and 3J). Combined, cardiac *Pacs2* ablation exacerbated MAM disruption and mitophagy reduction induced by HH.

### Hypobaric hypoxia reduced mitochondria-associated membrane formation and mitophagy *in vitro*

**Figure 4:**
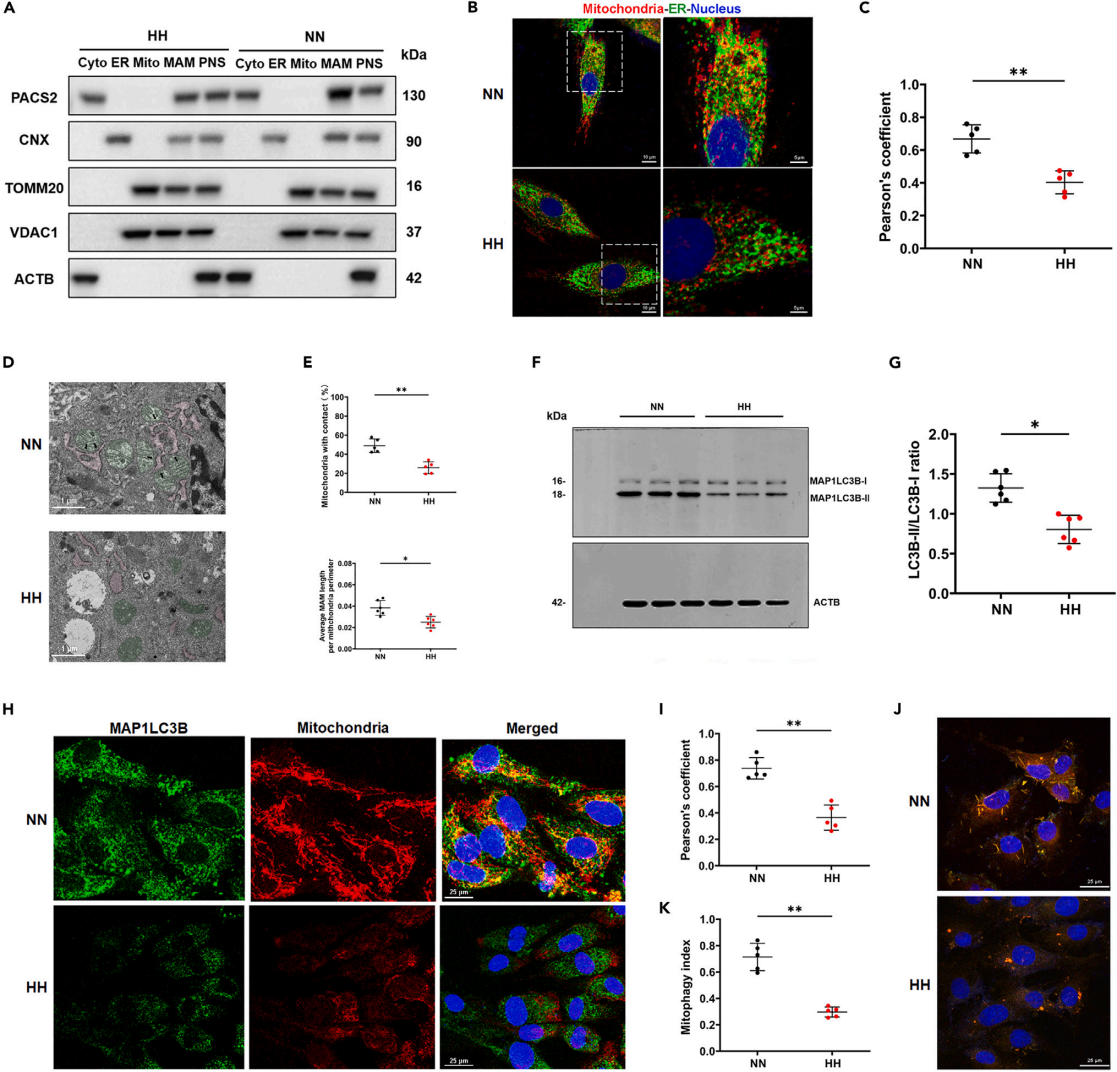
Hypobaric hypoxia reduced PACS2 expression, MAM formation, mitophagy, ER-mitochondria calcium flux, and mitochondrial oxidative phosphorylation *in vitro* (A) Western blot analysis of PACS2 and MAM-related proteins (CNX, TOMM2O, VDAC1) in H9C2 cardiomyocytes. (B) Cardiomyocytes marked by Mitotracker Deep Red and ERP72 (green) and then analyzed at higher magnification (Scale bar: 10 μm) and then analyzed at higher magnification (Scale bar: 5 μm). (C) Pearson’s overlap coefficient analysis indicates the lesser co-localization of ER with mitochondria in the HH group (N = 5 hearts per group). (D) Representative TEM images in HH-treated cardiomyocytes. Scale bar: 1 μm. ER are marked by red and mitochondria are marked by green. The black arrowheads indicate the ER-mitochondria contacts (<30 nm). (E) Quantification of the ratio (%) of mitochondria adjacent to ER (upper) and the average ER-mitochondria contacts (<30 nm) length per mitochondrion (below). (F and G) Representative western blots and statistical analysis (N = 6 hearts per group) for MAP1LC3B-I and MAP1LC3B-II under HH and NN exposures. (H and I) LSCM images of cardiomyocytes marked with Mitotracker Deep Red and MAP1LC3B (green). Merged images (H) and Pearson’s overlap coefficient analysis (I) revealed that HH exposure reduced the co-localization of MAP1LC3B and mitochondria (N = 5 hearts per group). Scale bar: 25 μm. (J and K) H9C2 cardiomyocytes were transfected by mtKeima-red for 12 h before HH exposure. LSCM images (J) and statistical analysis (K) showed decreased mitophagy index after HH exposure (N = 5 hearts per group). Scale bar: 25 μm. Data are shown as mean ± SD, ∗p < 0.05, ∗∗p < 0.01. CNX: calnexin; ER: ER HH: simulated hypobaric hypoxia; LSCM: laser scanning confocal microscopy; MAM: mitochondria-associated membranes; NN: simulated normobaric normoxia; TEM: transmission electron microscopy; TOMM20: translocase of the outer mitochondrial membrane member 20; VDAC1: voltage-dependent anion channel 1.

To explore the effect of HH on the MAM structure and biological function, we also measured the levels of MAM-related proteins in H9C2 cardiomyocytes exposed to simulated HH *in vitro*. As depicted in Figure 4A, in line with the *in vivo* results above, the expression of PACS2 in the MAM from HH-treated cells was lower than that in the MAM from NN-treated cells. Consistently, confocal imaging showed a decreased association between the ER and mitochondria in simulated HH-treated cardiomyocytes compared with that in the NN cells (Figures 4B and 4C). TEM imaging showed swollen mitochondria and fewer mitochondria adjacent to the ER after HH exposure (Figure 4D). With respect to the mitochondria-associated ER membranes, we also found a decrease in the ratio of close MAM contacts and relative length (Figure 4E). These data suggested that HH decreases the MAM junction structure in cardiomyocytes. Furthermore, we evaluated mitophagy levels in H9C2 cardiomyocytes. We found decreased MAP1LC3B-II transfer (Figures 4F and 4G) and co-localization with mitochondria after HH exposure (Figures 4H and 4I). To further verify the impaired mitophagy, we transfected the pH-dependent mitochondrial protein Keima into the cardiomyocytes; this can shift from green to red as mitochondria are delivered to lysosomes. Laser scanning confocal microscope (LSCM) monitoring showed that HH induced a markedly decreased mitophagy index in the cardiomyocytes (Figures 4J and 4K), indicating that HH decreased the number of mitophagosomes and impaired the mitophagy flux. The above results in the H9C2 cell lines as well show that HH reduces MAM formation and mitophagy.

### hypobaric hypoxia reduced endoplasmic reticulum-mitochondria calcium flux and mitochondrial oxidative phosphorylation *in vitro*

**Figure 5:**
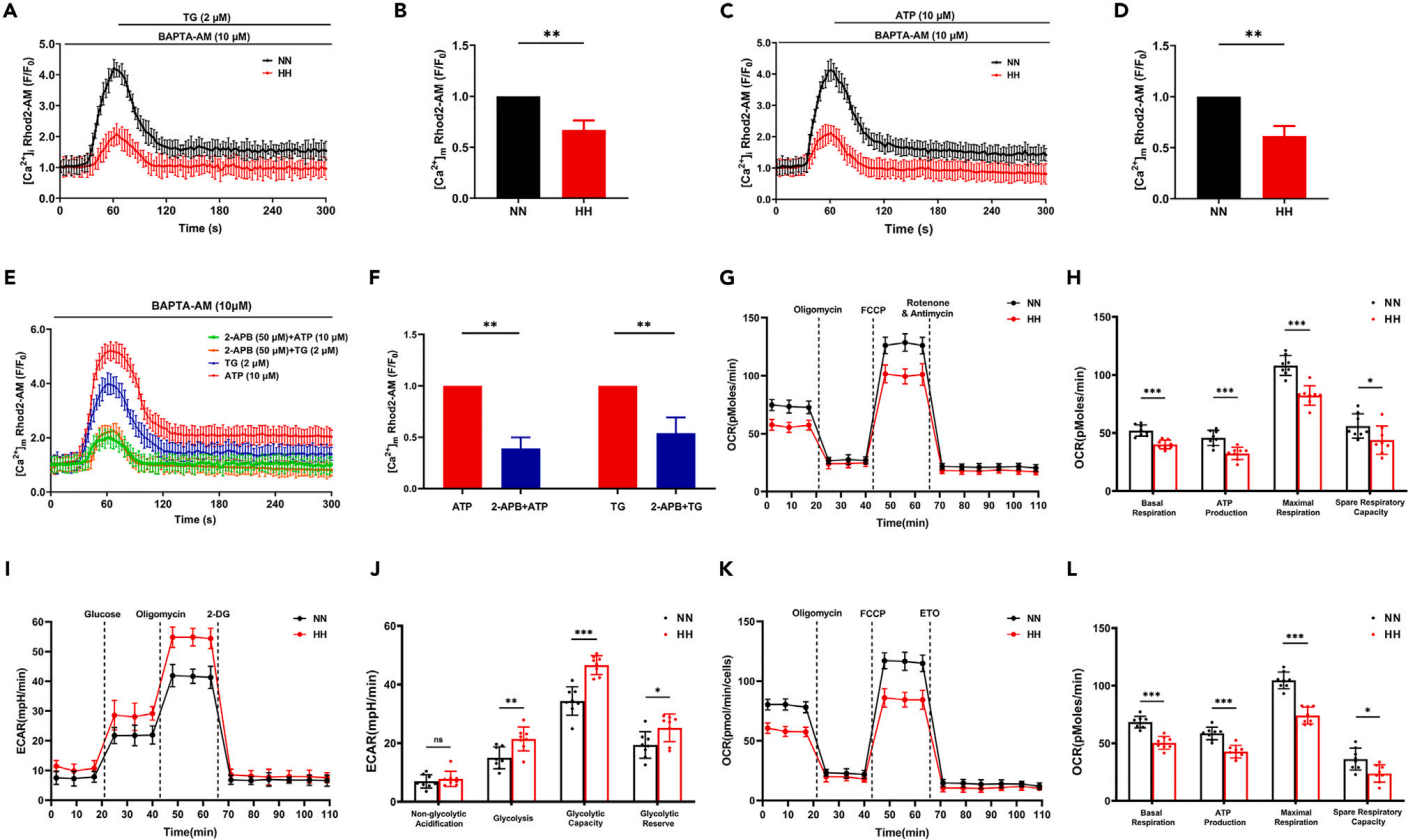
Hypobaric hypoxia reduced ER-mitochondria calcium flux and regulated mitochondrial energy metabolism *in vitro* (A-D) Cardiomyocytes from NN (black line) and HH groups (red line) are treated with BAPTA-AM (10 μM) for 30 min prior to and during exposure. Calcium in the mitochondria is stained by Rhod-2 AM probe. Images showing TG (2 μM) (A) and ATP (10 μM) (C) elicited calcium fluorescence signals. (B, D) Statistical analysis shows relatively lower ER-mitochondria calcium transfer in the HH group (cells in the NN group were used as controls). (E) Quantification of changes in mitochondria calcium concentration in cardiomyocytes pre-treated with 2-APB (50 μM) in NN condition. (F) Statistical analysis showing decreased mitochondrial calcium flux (cells without 2-APB treatment are used as controls). (G) The OCR and ECAR are determined with the Seahorse XF96 Extracellular Flux Analyzer. OCR is recorded at baseline and after the sequential injection of each compound (oligomycin, FCCP, and rotenone) at the indicated concentration. (H) Basal respiration, ATP production, maximal respiration, and spare respiratory capacity are calculated. (I) ECAR is recorded after the sequential injection of each compound (glucose, oligomycin, and 2-DG) at the indicated concentration. (J) Non-glycolytic acidification, glycolysis, glycolytic capacity, and glycolytic reserve are calculated. (K) OCR analysis of cells treated with BSA-conjugated palmitate substrate and after the sequential injection of each compound (oligomycin, FCCP, and ETO) at the indicated concentration. (L) Basal respiration, ATP production, maximal respiration, and spare respiratory capacity are calculated with FAO substrate. H9C2 cardiomyocytes are obtained from eight mice per group. Cells subjected with or without HH exposure before loading. Data are shown as mean ± SD, ∗p < 0.05, ∗∗p < 0.01. 2-APB: 2-aminoethoxydiphenyl borate; ATP: adenosine triphosphate; BAPTA-AM: bis-(aminophenolxy) ethane-N,N,N′,N′-tetra-acetic acid acetoxyme-thylester; ECAR: extracellular acidification rate; ETO: etomoxir; FCCP: trifluoromethoxy carbonyl cyanide phenylhydrazone; HH: simulated hypobaric hypoxia; NN: simulated normobaric normoxia; OCR: oxygen consumption rate; TG: thapsigargin.

PACS2 was reported to maintain the junction of the MAM and regulate mitochondrial calcium flux.^22^ In this study, we found that [Ca^2+^]_m_ in the H9C2 cardiomyocytes after HH treatment was markedly lower than in the NN group (Figures S4A and S4B). To determine the origin of mitochondrial calcium, we incubated H9C2 cardiomyocytes under HH or NN with a cytoplasmic Ca^2+^ chelator, BAPTA-AM (10 μM), in calcium-free Hanks' balanced salt solution (HBSS) for 10 min. Mitochondrial calcium was labeled using a Rhod2-AM probe, and the cells were observed and measured under LSCM. TG- (a calcium pump inhibitor, Figures 5A and 5B) and ATP- (an indirect IP3R agonist, Figures 5C and 5D) elicited ER-mitochondria calcium flux was lower in HH-treated cells than in NN-treated cells. Inositol trisphosphate receptors (IP3R) are important ER calcium-release channels.^27^ Therefore, we added 2-APB, which blocked the release of calcium from IP3R. Comparable mitochondrial calcium to HH exposure was observed when treated with 2-APB (Figures 5E and 5F), suggesting that IP3R is required for maintaining the physiological mitochondrial calcium levels under NN conditions. Next, we detected the effect of HH on the expression of two calcium transporters majoring in conveying calcium flux in the contact sites of ER and mitochondria, IP3R, and the mitochondrial calcium uniporter (MCU). As shown in Figures S3A and S3B, the expression of both two calcium channel proteins did not significantly change between NN and HH conditions. It is not the calcium transporters that limit the calcium flux across MAM, but the disruption of MAM contact was more likely to affect the calcium flux.

MAM formation and ER-mitochondrial calcium flux is essential for mitochondrial energy metabolism. Thus, we evaluated the effects of HH on cardiomyocyte mitochondrial energy metabolism using a Seahorse XF analyzer to measure mitochondrial respiration and glycolytic flux. We found that cardiomyocytes exhibited significant decreases in basal and maximal cellular oxygen consumption rate (OCR) in response to HH. ATP production and spare respiration capacity were also significantly lower after HH exposure (Figures 5G and 5H). ECAR results indicated an increase in glycolysis and glycolytic capacity owing to insufficient oxygen (Figures 5I and 5J). In addition, OCR measurement with a medium containing BSA-conjugated palmitic acid significantly decreased after HH exposure (Figures 5K and 5L). The above-mentioned results show that after HH exposure, the cardiomyocytes displayed metabolic reprogramming, represented by the restriction of FAO-related OXPHOS and a tendency to rely more on anaerobic than aerobic glycolysis for adapting to the HH condition. Since HH caused a decline in ER-mitochondria calcium flux, one mechanism that potentially accounts for the metabolic shift may be associated with the regulation of mitochondrial calcium.

### Endoplasmic reticulum-mitochondria calcium flux is involved in PACS2-mediated mitophagy and mitochondrial energy metabolism

**Figure 6:**
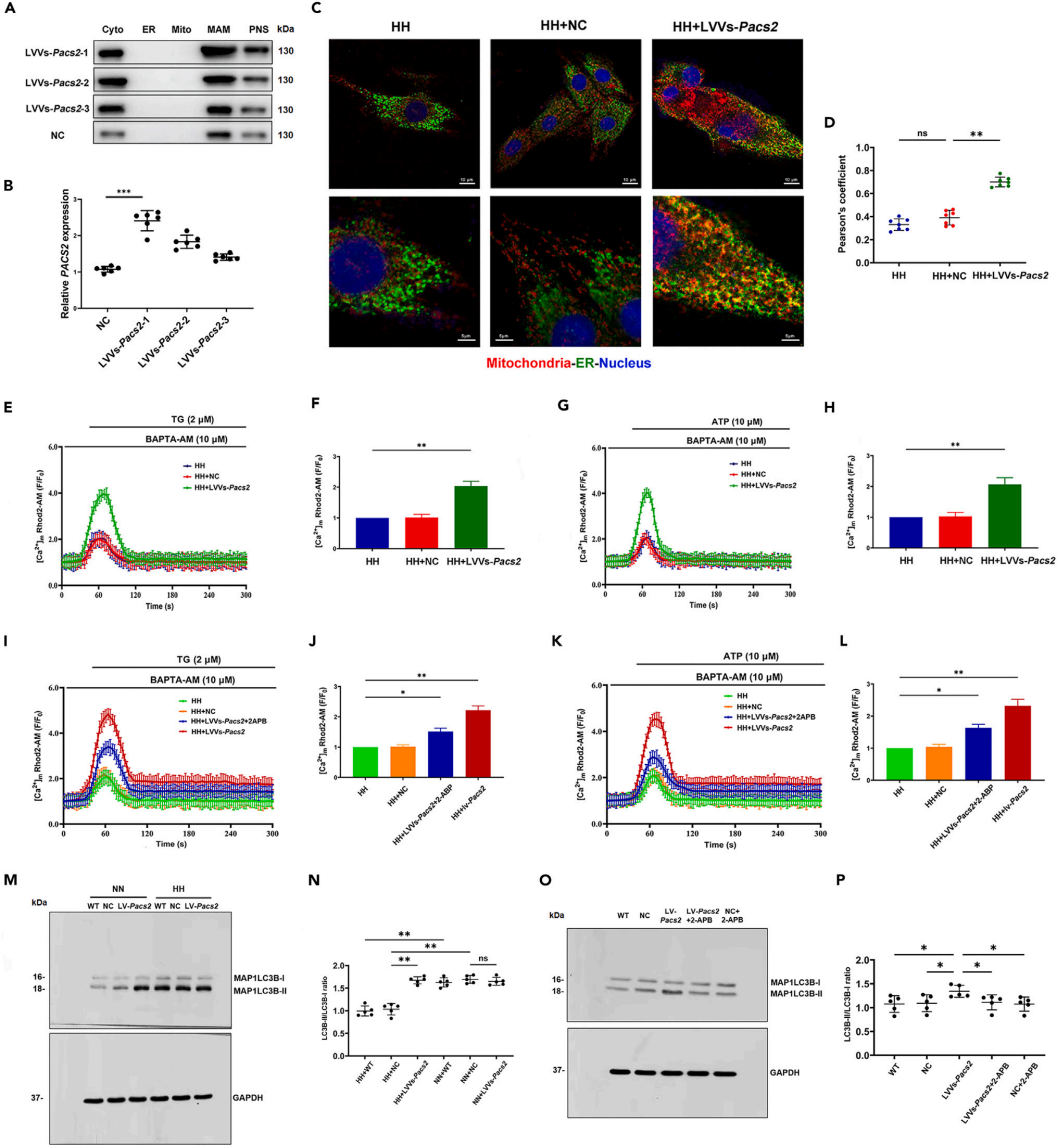
ER-mitochondria calcium flux is required for PACS2-mediated mitophagy (A and B) Western blot and statistical analysis showed that *Pacs2* is stably overexpressed in LVVs infected cardiomyocytes (N = 6 hearts per group). (C) Cardiomyocytes are co-immunostained for mitotracker (red) and ERP72 (green, Scale bar: 10 μm) and then analyzed at higher magnification (Scale bar: 5 μm). (D) Pearson’s overlap coefficient is employed to analyze the co-localization (N = 7 hearts per group). (E-H) Calcium in the mitochondria marked by Rhod-2 AM indicating reversed TG (E and F) and ATP (G and H) -mediated ER-mitochondria calcium fluorescence signals in *Pacs2* overexpression cells (green line) in HH condition (cells without 2-APB treatment are used as controls). (I-L) Mitochondria calcium evoked by LVVs overexpression of *Pacs2* (red line) in the presence of TG (I and J) and ATP (K and L) were blocked by 2-APB (blue line). (M and N) Western blots show significantly increased MAP1LC3B-II turnover in the LVVs-*Pacs2* group (N = 5 hearts per group). (M and N) Western blots show significantly increased MAP1LC3B-II turnover in the LVVs-*Pacs2* group (N = 5 hearts per group). (O and P) Western blots showing impaired PACS2-mediated MAP1LC3B-II turnover with the treatment of 2-APB (N = 5 hearts per group). Data are shown as mean ± SD, ∗p < 0.05, ∗∗p < 0.01. 2-APB: 2-aminoethoxydiphenyl borate; ATP: adenosine triphosphate; ER: ER HH: simulated hypobaric hypoxia; LVVs: lentiviral vectors; NN: simulated normobaric normoxia; TG: thapsigargin; NC: negative control.

We next determined the contributions of ER-mitochondria calcium in PACS2-induced mitophagy and mitochondrial energy metabolism. We obtained cardiomyocytes with stable overexpression of *Pacs2* through lentiviral vectors (LVVs) infection (Figures 6A and 6B). ER-mitochondria contacts increased in cells where *Pacs2* was overexpressed (Figures 6C and 6D). We found reversed [Ca^2+^]_m_ in LVVs*-*infected cardiomyocytes (Figures S4A and S4B). Similar results were observed with TG (Figures 6E and 6F) or ATP (Figures 6G and 6H) treatment in *Pacs2*-overexpressed cultured cells in the dynamics of mitochondrial calcium flux, indicating a source of calcium flux released from the ER. The regulation of calcium flux between the ER and mitochondria via IP3R is a major function of the MAM.^28^ As depicted in Figures 6I–6L, the restored calcium levels caused by the overexpression of *Pacs2* were partly blocked by 2-APB, suggesting that *Pacs2* overexpression promoted ER calcium release in the MAM through IP3R. Additionally, higher MAP1LC3B-II levels were observed after LVV-overexpression of *Pacs2* (Figures 6M and 6N), which could also be blocked by 2-APB (Figures 6O and 6P), suggesting that PACS2-mediated ER-mitochondria calcium flux was required for mitophagy.

**Figure 7:**
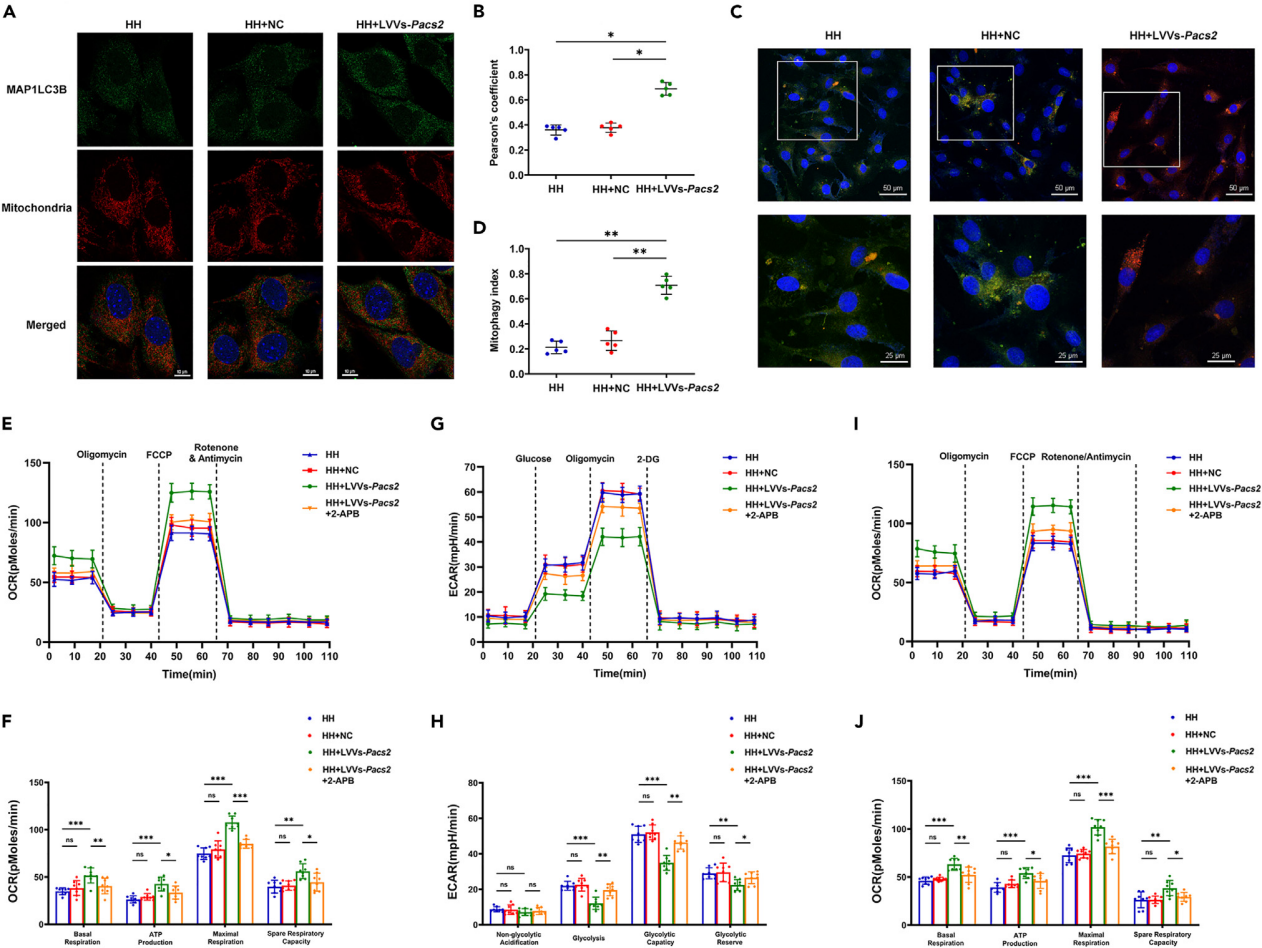
PACS2 supplementation alleviated impaired mitophagy and mitochondrial energy metabolism induced by HH (A and B) Mitotracker Deep Red and GFP-MAP1LC3B were used to mark H9C2 cardiomyocytes. Scale bar: 10 μm. Merged images (A) and Pearson’s overlap coefficient analysis (B) revealed that the overexpression of *Pacs2* increased the co-localization of MAP1LC3B-II and mitochondria during HH exposures (N = 5 hearts per group). (C and D) All three groups were infected by mtKeima plasmid for 12 h before HH exposure and observed under an LSCM. (C) Representative images showing puncta formation in three groups (Scale bar: 50 μm) and then analyzed at higher magnification (Scale bar: 25 μm). (D) Quantitative analysis of the fluorescent area showing increased mitophagy index in the LVVs-*Pacs2* group (N = 5 hearts per group). (E) Measurement of the OCR of cardiomyocytes (blue), negative controls (red), LVVs-*Pacs2* (green), and LVVs-*Pacs2*+2-APB (orange) in HH exposure. (F) Basal respiration, ATP production, maximal respiration, and spare respiratory capacity are calculated. (G) Measurement of the ECAR of cardiomyocytes (blue), negative controls (red), LVVs-*Pacs2* (green), and LVVs-*Pacs2*+2-APB (orange) in HH exposure. (H) Non-glycolytic acidification, glycolysis, glycolytic capacity, and glycolytic reserve are calculated. (I) OCR analysis of cells treated with BSA-conjugated palmitate substrate and after the sequential injection of each compound (oligomycin, FCCP, and ETO) at the indicated concentration. (J) Basal respiration, ATP production, maximal respiration, and spare respiratory capacity are calculated with FAO substrate. H9C2 cardiomyocytes are obtained from eight mice per group. Data are shown as mean ± SD, ∗p < 0.05, ∗∗p < 0.01. 2-APB: 2-aminoethoxydiphenyl borate; ATP: adenosine triphosphate; BAPTA-AM: bis-(aminophenolxy) ethane-N,N,N′,N′-tetra-acetic acid acetoxyme-thylester; ECAR: extracellular acidification rate; ETO: etomoxir; FCCP: trifluoromethoxy carbonyl cyanide phenylhydrazone; HH: simulated hypobaric hypoxia; LSCM: laser scanning confocal microscopy; NN: simulated normobaric normoxia; OCR: oxygen consumption rate; TG: thapsigargin.

With the supplementation of PACS2, more MAP1LC3B puncta co-localized with mitochondria (Figures 7A and 7B) and an increased mitophagy index were observed in HH conditions (Figures 7C and 7D). These data showed that PACS2 restored impaired mitophagy through enhanced ER-mitochondria calcium flux. To investigate whether ER-mitochondria calcium flux was also involved in PACS2-mediated mitochondrial energy metabolism alteration, we compared real-time changes in OCR and ECAR in the H9C2 cardiomyocytes with or without overexpression of PACS2 under HH treatment. With PACS2 supplementation, the decreased basal respiration, ATP production, and maximal respiration (Figures 7E and 7F) and increased basal and maximal ECAR (Figures 7G and 7H) induced by HH were significantly reversed. The recovery in mitochondrial respiration was also blocked by 2-APB treatment (Figures 7G and 7H). To extend the hypothesis that PACS2 helped recover OCR, which is supported by FAO, we further measured OCR in a medium containing palmitate-BSA as an exogenous FAO substrate. Notably, the cardiomyocytes showed a reversed OCR after the supplementation of PACS2 when compared with an empty vector control (Figures 7I and 7J), which was significantly blocked on adding 2-APB. This indicates that PACS2 enabled HH-treated cardiomyocytes to switch from glycolysis to an increased reliance on FAO for ATP production. This metabolic reprogramming at least partly depends on the calcium flux across MAM. Together, these data suggested that ER-mitochondria calcium flux was essential for PACS2-mediated mitophagy maintenance and mitochondrial energy metabolism after HH exposure.

### Cardiac *Pacs2* knock-in alleviated hypobaric hypoxia-induced right cardiac dysfunction

**Figure 8:**
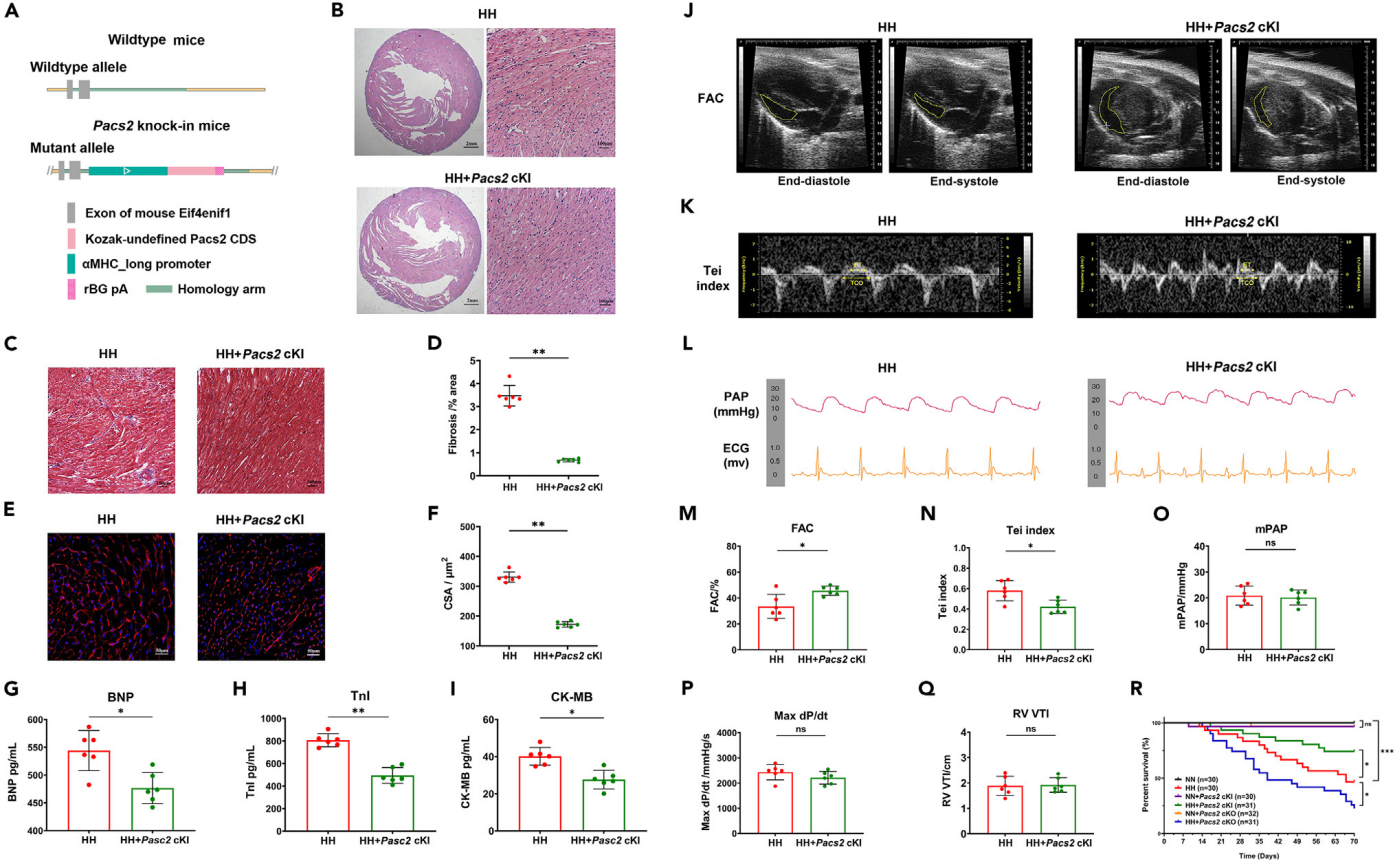
Cardiac *Pacs2* knock-in alleviated HH-induced right myocardium injury and right cardiac dysfunction (A) Schematic of *Pacs2* cKI mice model. (B and C) Representative photographs of HE staining (B) and Masson’s trichrome staining (C) of the right myocardium in *Pacs2* cKI mice heart and littermate controls. Scale bar: 100 μm. (D) Quantification of fibrotic area revealing less myocardial fibrosis area (blue) in *Pacs2* cKI mice (N = 6 hearts per group). (E) Representative images of the right myocardium stained with WGA (red) to delineate sarcolemma and DAPI (blue). Scale bar: 50 μm. (F) Bar graphs revealing cardiomyocytes CSA in the two groups (N = 6 hearts per group). (G-I) Statistics of plasma concentration of BNP, TnI, and CK-MB of *Pacs2* cKI mice and controls during HH exposure (N = 6 hearts per group). (J) FAC measurement of the RV in the control group (FAC = 28.51%) and *Pacs2* cKI mice group (FAC = 50.68%) in HH exposure. Representative images acquired at end-diastole (left) and end-systole (right). (K) Tei index was measured in the control group (Tei index = 0.57) and *Pacs2 Pacs2* cKI mice group (Tei index = 0.34). (L) mPAP measured by RHC and the ECG of the control mice group (mPAP = 22.58 mmHg) and *Pacs2* cKI mice group (mPAP = 21.67 mmHg). (M-Q) Statistics of FAC (M), Tei index (N), mPAP (O), max dP/dt (P), RV VTI (Q) in the two groups (N = 6 hearts per group). (R) Survival rate of the control and modeling mice with or without HH exposure. Right myocardium is obtained from six mice per group, data are shown as mean ± SD, ∗p < 0.05, ∗∗p < 0.01. BNP: brain natriuretic peptide; CK-MB: creatine kinase MB; CSA: cross-sectional area; FAC: fractional area change; HH: hypobaric hypoxia; mPAP: mean pulmonary artery pressure; NN: normobaric normoxia; RHC: right cardiac catheterization; RV: right ventricular; TnI: troponin I; VTI: velocity time integral; WGA: wheat germ agglutinin.

LVVs-overexpression of *Pacs2* significantly reversed MAM formation, mitophagy, and mitochondrial energy metabolism in cardiomyocytes *in vitro*. To verify the contributions of PACS2 in maintaining RV function during HH exposure *in vivo*, we generated cardiomyocyte-specific *Pacs2* knock-in mouse models (*Pacs2* cKI; Figure 8A). Histological analysis of the hearts from the *Pacs2* cKI mice showed significantly decreased right cardiac hypertrophy (Figure 8B), cardiac fibrosis area (Figures 8C and 8D), and cardiomyocytes CSA (Figures 8E and 8F) with HH exposure. In addition, the *Pacs2* cKI mice had lower plasma levels of BNP, TnI, and CK-MB (Figures 8G–8I) than their littermate controls, indicating that PACS2 supplementation reduced the HH-induced myocardial damage. Compared with their littermate controls, the *Pacs2* cKI mice exhibited a higher RV FAC (Figures 8J and 8M) and lower Tei index (Figures 8K and 8N). As expected, *Pacs2* overexpression did not significantly alter mPAP, max dP/dt, and RV VTI (Figures 8O–8Q) during HH exposure. *Pacs2* cKI did also fail to completely reverse the impaired cardiac function to the baseline level as the NN group, as shown in Figure S5. Additionally, in the NN condition, *Pacs2* cKI had little effect on the normal right cardiac function, representing by no significant difference in FAC, Tei index, mPAP, et al. when compared with their littermate controls. The survival of mice without HH exposure were higher than those in HH exposure groups. Compared with WT mice without HH exposure, survival rate was significantly increased in the *Pacs2* cKI group while decreased in the *Pacs2* cKO group (Figure 8R). These data suggested that conditional *Pacs2* cKI reduced cardiomyocyte injury and partially recovered RV cardiac function after HH exposure without significantly influencing the RV afterload. With the progressive right cardiac injury caused by HH, the left cardiac function in mice has also been affected significantly. We observed a significant increase in the left ventricular dimensions (end-systolic diameter and end-diastolic diameter) and a significant decrease in the systolic function (left ventricular ejection fraction and left ventricular fractional shortening) in the mice with HH exposure compared to the NN group (Figures S6A–S6D). Moreover, the effect of PACS2 genetic manipulation appeared to be significant in altering the left cardiac function, as shown by aggravated left cardiac dysfunction caused by *Pacs2* cKO and alleviated HH-induced left cardiac dysfunction in *Pacs2* cKI mice (Figures S6A–S6D).

## Discussion

This is the first study to reveal the underlying mechanism of PACS2 in HH-mediated cardiomyocyte injury and right cardiac dysfunction. The core process was the downregulated PACS2 localized in the MAM after HH exposure. PACS2 reduction further suppressed MAM formation and resulted in decreased calcium flux from the ER to the mitochondria via the IP3R calcium channel. The reduced mitochondrial calcium influx further inhibited mitophagy and mitochondrial energy metabolism, inducing cardiomyocyte injury and right cardiac dysfunction. Moreover, cardiomyocyte-specific knock-in of *Pacs2* reversed right cardiac dysfunction and RV fibrosis. Of note, neither conditional cKO nor cKI of the *Pacs2* in cardiomyocyte influenced the RV afterload, highlighting an independent role of PACS2-directed cardiomyocyte responses in maintaining right cardiac function. Thus, our results provided potential therapeutic targets for high-altitude-induced right cardiac impairment.

Sufficient oxygen supply is the most essential condition for the survival and function of cardiomyocytes. Hypoxia induces pulmonary vasoconstriction and increases pulmonary vascular resistance. Prolonged hypoxia further results in right cardiac function impairment and even right heart failure.^29^ Thus, in this study, we focused on the right rather than on the left cardiac function. Hypoxia is usually generated by normobaric hypoxia (NH) or HH in the experiments. NH lowers the partial pressure of inspired oxygen (PiO_2_) by reducing the fraction of inspired oxygen by adding exogenous nitrogen without altering the barometric pressure. Conversely, HH lowers the PiO_2_ by reducing the barometric pressure. Previous studies have suggested that NH and HH induced similar cardiac adaptations over a short duration, although lower SpO_2_ and worse right cardiac function emerged during long-term exposure.^30^^,^^31^ Thus, more complicated mechanisms may exist in HH than in NH, including intravascular bubble formation, increased alveolar dead space, altered fluid permeability, and a mismatch in ventilation and perfusion.^32^ Our study established a long-term HH exposure model to simulate real cardiac function alteration and cardiomyocyte response in high-altitude environments. Previous studies on cardiomyocyte injury caused by hypoxia mainly focused on the increase in oxygen free radicals and anaerobic metabolites, eventually leading to cardiomyocyte apoptosis, myocardial fibrosis, and irreversible cardiac remodeling.^33^ Our study indicated that both mitophagy and mitochondrial energy metabolism were involved in cardiomyocytesurvival under HH conditions. Our results revealed a mechanism that results in right cardiac dysfunction at high altitudes.

Previous studies reported that PACS2 is closely associated with the onset and progression of tumors, such as colorectal and liver cancer.^34^^,^^35^ Owing to their infinite proliferation ability, tumor cells survive in relative hypoxic conditions. Therefore, it is essential to understand the biological function of PACS2 in regulating cell fate in response to hypoxic conditions. Accordingly, after HH exposure, PACS2 expression was found to be markedly reduced in the MAM, although with a moderate increase in PACS2 in the cytosol both *in vivo* and *in vitro* (Figures 4A and 5A). This implied a dynamics translocation from the MAM to the cytosol, which may partly explain the decreased PACS2 in the MAM. The free form of PACS2 in the cytosol contributed to nuclear gene expression and membrane trafficking rather than calcium flux, thus exhibiting the suppression of downstream mitophagy and energy metabolism. Besides PACS2, recent studies have found that the FUN14 domain containing 1 (FUNDC1), a new protein in the MAM, is responsible for the release of calcium from the ER to the mitochondria and mitophagy induction in mouse cardiomyocytes.^36^^,^^37^ Notably, our results revealed that PACS2 served as a mitophagic regulator in the MAM and modulated calcium release from ER to mitochondria in cardiomyocytes. However, the precise molecular mechanism of how PACS2 cooperates with FUNDC1 to regulate calcium flux, mitophagy, and cardiac function remains unknown. We considered that a few special proteins in the MAM may interact to form a protein complex or “protein machine” and involve themselves in the above-mentioned process. PACS2 may be the key protein and serves as a scaffold to sponge other proteins.

Mitophagy is essential for mitochondrial homeostasis and quality control in cardiomyocytes.^38^ During hypoxia, mitophagy is the sole mechanism through which cardiomyocytes eliminate superfluous or damaged mitochondria.^14^ However, the mechanisms underlying mitophagy remain largely unknown. Previous studies on mitophagy have focused on several protein receptors on the mitochondrial membrane, including BCL2 interacting protein 3 (BNIP3), BNIP3-like, and FUNDC1. Most of them have a classic LIR motif to directly bind MAP1LC3B for mitophagy activation.^39^ In this study, we found a new protein in the MAM without this classic LIR motif, although it was closely associated with HH-mediated mitophagy. PACS2 did not directly link to autophagy-associated proteins, such as ATG5, ATG7, Beclin1, and MAP1LC3B; however, it acted as a calcium channel to promote calcium influx into the mitochondria.^22^ Usually, intracellular calcium is considered an activator of autophagy.^40^^,^^41^^,^^42^

To date, the role of calcium signaling in autophagy regulation is highly controversial. Most studies considered that calcium works as an autophagy activator because calcium mobilizing agents and calcium ionophores promote autophagy by elevating intracellular calcium concentration.^43^ In this study, we detected that PACS2-mediated calcium influx was required for HH-induced mitophagy in cardiomyocytes, which further verified the effect of intracellular calcium on mitophagy regulation. However, the mechanism through which mitochondrial calcium is involved in mitophagy activation requires further exploration. Mitochondrial calcium uptake occurs mostly through MAM, which closely contacts with the ER and renders a micro-domain with a sufficiently high calcium concentration.^44^ A recent study reported that mitochondrial calcium influx inhibition decreased ATP production, enhanced mitophagy, and provided cardioprotection in cardiac failure.^45^ Conversely, we found that HH decreased ER-mitochondria calcium flux and calcium-mediated mitophagy. Similar to our results, Böckler and Zou demonstrated that ER-mitochondria contact and calcium flux across the MAM were required for autophagic removal of mitochondria since artificially tethering ER and mitochondria rescued mitophagy defects.^18^^,^^33^ Besides mediating calcium flux, the ER-mitochondria encounter structure may also supply the growing phagophore with lipids synthesized in the ER, which then enclose the impaired mitochondria to form a mitophagosome. Hence, ER-mitochondria-mediated calcium flux is required for mitophagy induction.

In addition to the above-mentioned role of calcium in mitophagy, mounting evidence suggests that calcium also dynamically regulates the aerobic energy metabolism by stimulating mitochondrial OXPHOS.^46^^,^^47^ In highly energy-consuming tissues, such as the heart, OXPHOS in the mitochondria provides a major source of cellular ATP through the oxidation of substrates, including fatty acids, glucose, and ketones.^48^ We found that to adapt to the HH condition, cardiomyocytes mainly rely on the glycolytic pathway rather than the OXPHOS pathway. As a critical signaling molecule in mitochondrial energy conversion, sufficient mitochondrial calcium concentration is required to activate mitochondrial dehydrogenases, including the pyruvate dehydrogenase complex (PDHC), NADH-isocitrate dehydrogenase (ICDH), and α-ketoglutarate dehydrogenase (α-KGDH).^49^^,^^50^ Other components within the energy-producing pathways besides NADH generation, such as downstream ATPase and the cytochrome chain, were also significantly stimulated by calcium.^51^^,^^52^ Current studies supported the proposed physiological metabolic role of calcium entry into the mitochondria matrix through the mitochondrial calcium uniporter (MCU) complex.^53^ Together with some recent reports, we indicated that the IP3R channels were also associated with alterations in mitochondrial calcium flux, especially in cardiomyocytes under HH exposure. IP3R-mediated calcium signaling required quantification of PACS2 and proximity of the ER and mitochondria. The supplement of PACS2 could improve mitochondrial respiration efficiency during HH exposure. Our previous randomized double-blinded clinical trial proposed that cardiac function could be recovered by optimizing myocardial energy metabolism.^54^ Combined with those results, our present results provide a therapy for improving cardiac function at high altitudes targeted on energy metabolic reprogramming based on calcium flux across the MAM in cardiomyocytes. Moreover, linking reprogramming of energy metabolism induced by the PACS2 supplement was associated with enhanced mitophagy. We considered that mitophagy may, at least partly, provide relatively efficient substrates such as fatty acids for maintaining energy demand. However, the exact role of calcium in cardiomyocyte energy metabolism reprogramming requires confirmation using accurate techniques such as isotope tracing analysis.

In conclusion, we described acardiomyocyte injury mechanism during HH exposure in high-altitude environments. HH downregulated the expression of PACS2 in the MAM. Decreased PACS2 disrupted MAM formation and calcium transfer from the ER to the mitochondria, leading to mitophagy inhibition and mitochondrial energy metabolism impairment, which induced cardiomyocyte injury and right cardiac dysfunction during HH exposure. Our study identified a potential target for the prevention and treatment of cardiovascular diseases caused by high-altitude exposure.

### Limitations of the study

Due to the limitations in the present study, we did not explore the possible molecular mechanisms that contribute to the downregulation of PACS2 in cardiomyocytes under HH conditions. Besides, MAM is also abundant and important in the left ventricular,^19^^,^^36^ therefore further studies are warranted to investigate the different chamber’s expression of PACS2 response to HH exposure and its role in modulating the cardiac function. Finally, there were no human samples from patients. The clinical relevance of the above findings needs further validation in the future.

## STAR★Methods

### Key resources table

**Table:** 

REAGENT or RESOURCE	SOURCE	IDENTIFIER
**Antibodies**		

Rabbit polyclonal anti-PACS2	Abcam	Cat# ab222316
Rabbit monoclonal anti-Mitofusin 2	Abcam	Cat# ab124773
Mouse monoclonal anti-VDAC1	Abcam	Cat# ab14734
Rabbit monoclonal anti-FACL4	Abcam	Cat# ab155282
Rabbit monoclonal anti-TTC11/FIS1	Abcam	Cat# ab156865
Rabbit monoclonal anti-Calnexin	Abcam	Cat# ab133615
Rabbit monoclonal anti-LC3B	Abcam	Cat# ab192890
Rabbit monoclonal anti-TOMM20	Abcam	Cat# ab186735
Mouse monoclonal anti-beta Actin	Abcam	Cat# ab8226
Rabbit polyclonal anti-ERp72	Abcam	Cat# ab155800

**Bacterial and virus strains**		

LVVs carrying Pacs2 RNA system	Gene Pharma Technology	N/A

**Chemicals, peptides, and recombinant proteins**		

Bafilomycin A1	Sigma-Aldrich	Cat#B1793
TG	Sigma-Aldrich	Cat#T9033
adenosine triphosphate	Sigma-Aldrich	Cat# A1852
2-bis (2-aminophenoxy) ethane-N,N,N’,N’-tetraacetic acid tetrakis	Molecular probes	Cat#B1205
2-aminoethoxydiphenyl borate	Abcam	Cat#ab120124
Tris-buffered saline	Boster Biological Technology	Cat#AR0031
Tween-20	Solarbio	Cat#T8220
Dulbecco’s modified Eagle’s medium	Sigma-Aldrich	Cat#0030034DJ
Fetal bovine serum	Hy Clone Laboratories	Cat#SV30208.03
Rhod2 AM	Abcam	Cat#ab142780
MitoTracker Deep Red	Invitrogen	Cat#M22426

**Critical commercial assays**		

BNP ELISA kit	Jiangsu Jingmei Biological Technology	Cat#JM-02343M2
TnI ELISA kit	Jiangsu Jingmei Biological Technology	Cat#JM-02662M2
CK-MB ELISA kit	Jiangsu Jingmei Biological Technology	Cat#JM-03084M2
XF Cell Mito Stress Test Kit	Seahorse Bioscience	Cat#103015-100

**Deposited data**		

iTRAQ proteomics analysis/nanoUHPLC-MS/MS analysis data	This paper	Table S1, iProX database; https://www.iprox.org (Project ID: IPX0005958000)
LC-MS metabolomics data	This paper	Table S2

**Experimental models: Cell lines**		

Rat: H9C2 cardiomyocytes	Chinese Academy of Sciences	BFN60804388

**Experimental models: Organisms/strains**		

Mouse: C57BL/6J, *Pacs2* cKO(Pacs2flox/flox/CreαMHC+/−)	Cyagen Biosciences	N/A
Mouse: C57BL/6J, *Pacs2* cKI	Cyagen Biosciences	N/A
Mouse: PACS2fl/fl	Cyagen Biosciences	N/A
Mouse: Cre transgenic mice	Cyagen Biosciences	N/A

**Recombinant DNA**		

Plasmid: mitochondria-targeted monomeric Keima-Red-hyg	Medical and Biological Laboratories	Cat#AM-V0251HM

**Software and algorithms**		

Image-Pro Plus 5.0	GE Healthcare	https://imagej.nih.gov/ij
LAS X software	Leica	https://www.leica-microsystems.com/products/microscope-software
LabChart 7 software	ADInstruction	https://www.adinstruments.com.cn/products/labchart
Seahorse XF Extracellular Flux analyzer software	Agilent	https://www.agilent.com.cn/zh-cn/product/cell-analysis/real-time-cell-metabolic-analysis/xf-software/seahorse-wave-desktop-software-740897

### Resource availability

#### Lead contact

Further information and requests for resources and reagents should be directed to and will be fulfilled by the lead contact, Lan Huang (lhuang@tmmu.edu.cn).

#### Materials availability

This study did not generate new unique reagents.

### Experimental model and subject details

#### Development of a chronic HH-induced mouse model

All animal procedures were approved by the Experimental Animal Ethics Committee of the Army Medical University and conformed to the regulations of the Guide for the Care and Use of Laboratory Animals. Male C57BL/6J mice (6–8 weeks old) were housed in a temperature-controlled environment with a 12-hour light/dark cycle and had free access to water and food. For HH exposure, mice were subjected to a high-altitude low-pressure chamber (Fenglei Aviation Ordinance Co., Ltd, Guizhou, China) with reduced ambient air pressure to simulate an environment of high altitude of 5000 m (HH, approximately 424 mmHg, or the equivalent of 11.0% O_2_) for 6 weeks. Control mice were raised at an altitude of the sea level (NN, approximately 760 mmHg, or the equivalent of 20.9% O_2_) out of the hypobaric chamber for 6 weeks (Figure 1A).

#### Generation of cardiomyocyte-specific *Pacs2* knockout mice

Cardiomyocyte-specific *Pacs2* knockout (*Pacs2* cKO*)* mice were generated on a C57BL/6J background by the CRISPR/Cas9 system at Cyagen Biosciences. The gRNA to mouse *Pacs2* gene, the donor vector containing loxP sites, and Cas9 mRNA were co-injected into fertilized mouse eggs to generate targeted conditional knockout offspring. *Pacs2* ^*flox/flox*^ mice in which the *Pacs2* gene was flanked by loxP sites within introns 1 and 3 (KO region: approximately 1842 bp) were crossed with α-myosin heavy chain (αMHC) promoter-Cre transgenic mice (Cyagen Biosciences) to obtain *Pacs2*^*flox/+*^*/Cre*^*αMHC+/−*^ mice. F0 founder animals were identified by polymerase chain reaction (PCR) followed by sequence analysis, which were bred to wild-type mice to test germline transmission and F1 animal generation. F1 founders, including *Pacs2* cKO (*Pacs2*^*flox/flox*^*/Cre*^*αMHC+/−*^) mice, were genotyped by tail genomic PCR.

#### Generation of cardiomyocyte-specific *Pacs2* knock-in mice

The *Pacs2* cKI in C57BL/6J mice was created using CRISPR/Cas-mediated genome engineering (Cyagen Biosciences). The Hipp11 locus is located within an intergenic region between the *Eif4enif1* and *Drg1* genes on mouse chromosome 11. The mouse *Pacs2* gene (NCBI Reference Sequence: NM_001291444.1) is located on mouse chromosome 12. For the KI model, the ‘alphaMHC_long promoter-Kozak-Mouse *Pacs2* CDS-rBG pA’ cassette was inserted into the Hipp11 locus (approximately 0.7 kb 5' of Eif4enif1 gene and 4.5 kb 3' of the Drg1 gene). To engineer the targeting vector, homology arms were generated by PCR using a BAC clone as the template. Cas9 and gRNA were co-injected into fertilized eggs with a targeting vector for mice production. The pups were genotyped by PCR followed by sequencing analysis.

### Method details

#### Blood preparation and enzyme-linked immunosorbent assay

Detection kits for mouse plasma BNP, troponin I (TnI), and creatine kinase MB (CK-MB) were purchased from Jiangsu Jingmei Biological Technology Co., Ltd. (Jiangsu, China). Approximately 1.5 mL of blood was drawn from each mouse and stored in procoagulant tubes. Plasma was separated by centrifugation (3000×g, 20 minutes) after coagulation at room temperature for 10 minutes. The plasma levels of BNP, TnI, and CK-MB were measured using a commercially available BNP enzyme-linked immunosorbent assay (ELISA) kit (JM-02343M2, 210727B8), TnI ELISA kit (JM-02662M2, 210727I4), and CK-MB ELISA kit (JM-03084M2, 210727C6), respectively, following the manufacturer’s instructions.

#### Hemodynamic monitoring

RHC was performed using a pressure detecting device (ADInstruments Mikro-Tip®, MPVS Ultra RSBMIL002/M) after a 6-week HH or NN exposure. The mice were placed on a heated pad and anesthetized with 2% isoflurane. The right jugular vein was exposed, and a 1F needle (ADInstruments Mikro-Tip®, SPR-1000) was slightly bent inwards to conduct the cannula containing the catheter into the jugular vein. The cannula was maneuvered to the right ventricle, with its tip pointing toward the heart until an RV pressure curve could be identified using LabChart 7 software. Subsequently, the cannula tip was manipulated to the left and superiorly. The catheter was advanced into the main pulmonary artery, passing through the pulmonary valve. When the catheter enters the main pulmonary artery, the diastolic pressure rises on the monitor, and a pulmonary artery pressure curve appears. When the curve was constant, the related indices, such as the mean pulmonary artery pressure (mPAP), the maximum positive time derivative of left ventricular pressure (max dP/dt), and RV VTI and electrocardiograms were measured.

#### Evaluation of RV hypertrophy

After the hemodynamic measurement, the mice were sacrificed by cervical dislocation, and their hearts were removed quickly and weighed. The free wall of the right ventricle was dissected from the left ventricle and interstitial septum. Whole heart weight (normalized by body weight) and Fulton’s index (right ventricle / [left ventricle + interstitial septum]) were used as indices of cardiac hypertrophy.

#### Histological analysis

The hearts from the mice exposed to NN and HH were excised, placed in 4% paraformaldehyde, dehydrated in graded concentrations of ethanol, immersed in xylene, and embedded in paraffin. Sections of 5-μm thickness were cut on a microtome with a disposable blade, stained with HE and Masson’s trichrome stains, and examined by light microscopy. The cardiomyocyte CSA was analyzed by staining the heart sections with a wheat germ agglutinin–Alexa Fluor® 647 conjugate (W32466, Invitrogen). Six mice from each group were included in the histological analysis. A minimum of five cross-sections of each heart were examined, and the measurements were averaged for statistical analysis. ImageJ software (RRID:SCR_003070) was used to quantify all the histological endpoints.

#### Echocardiography

Cardiac geometry and function were examined using ultrasonography (GE Vivid 7 Dimension, L15/6-MHz transducer). The mice were anesthetized with 2% isoflurane while maintaining proper body temperature (36–37°C) and heart rate (450–550 beats/ minute). The temporal frame rate in the echo mode was set to 60 Hz. A 1.0-mm sampling gate was used to obtain the inflow and outflow velocities, and the maximal sweep speed was 200 mm/s. RV end-diastolic (ED), and end-systolic (ES) areas were measured using ImageJ from the apical or basal four-chamber views at end-diastole or end-systole. The RV FAC was calculated as follows: FAC = ([ED RV area – ES RV area] / ED RV area) × 100%. For Tei index calculation, the tricuspid closure opening time (TCO) and ejection time (ET) were measured from tissue Doppler myocardial velocity images, as follows: Tei index = (TCO – ET) / ET. Data were collected from six mice per group and represented the average of a minimum of five separate scans in a random blind fashion. To avoid bias, the researcher performed all echocardiography procedures blinded to the experimental treatments.

#### Western blotting

Cardiomyocyte MAM fractions *in vitro* and MAM fractions of the hearts were isolated following a previously described protocol. Western blotting was used to evaluate protein expression in different fractions. Briefly, the protein concentrations of different fractions after isolation were detected using the bicinchoninic acid (BCA) assay (Beyotime Biotechnology, P0012). The same mass of total protein was separated by sodium dodecyl sulfate–polyacrylamide gel electrophoresis and transferred to polyvinylidene fluoride membranes (Millipore). The membranes were blocked with 5% non-fat milk in Tris-buffered saline (Boster Biological Technology, AR0031) containing 0.5% Tween-20 (Solarbio, T8220), and membrane-bound proteins were probed with primary antibodies purchased from Abcam against the following antigens: PACS2 (ab222316, Abcam), mitofusin 2 (MFN2; ab124773, Abcam), voltage-dependent anion-selective channel protein 1 (VDAC1; ab14734, Abcam), acyl-CoA synthetase 4 (FACL4; ab92501, Abcam), mitochondrial fission 1 (FIS1; ab156865, Abcam), calnexin (ab133615, Abcam), microtubule-associated protein 1 light chain 3 beta (MAP1LC3B; ab192890, Abcam), translocase of outer mitochondrial membrane 20 (TOMM20; ab186735, Abcam), and actin beta (ACTB; ab8226, Abcam). Protein bands were visualized by chemiluminescence detection and quantified using the Image QuantTL software (GE Healthcare, Sweden).

#### Cell culture and RNA transfection

Rat H9C2 cardiomyocytes (BFN60804388) were purchased from the Cell Bank of the Chinese Academy of Sciences (Shanghai, China). Cardiomyocytes were cultivated in Dulbecco’s modified Eagle’s medium (Sigma-Aldrich, Louis, MO, USA) and 10% fetal bovine serum (Hy Clone Laboratories, PA, USA) and supplemented with 1% antibiotic-antimycotic (1000 U/mL penicillin and 100 μg/mL streptomycin). H9C2 cardiomyocytes in the NN group were incubated at 37°C with 5% CO_2_. HH conditions were achieved by the hypobaric chamber (Billups-Rothenberg) -simulated high-altitude environment, which was flushed with a pre-analyzed gas mixture of 1% O_2_, 5% CO_2_, and 94% N_2_. To maintain cardiomyocyte cultures, the medium was changed every two days. LVVs carrying *Pacs2* RNA system were constructed by Gene Pharma Technology (Shanghai, China). The LVVs were added to the cells at a multiplicity of infection of 100. The transfection medium was changed two days later, and the cells were continuously cultured in fresh medium. Real-time quantitative reverse transcription-PCR and western blotting were used to detect the efficiency of *Pacs2* overexpression in cardiomyocytes.

#### Measurement of mitochondrial calcium in intact cells

Cardiomyocytes were seeded on glass-bottomed cell culture dishes and incubated with 1 μM of the calcium indicator Rhod2-AM (ab142780, Abcam) at 37°C in the dark for 30 minutes, as per the manufacturer’s guidelines. Subsequently, the cells were washed twice with calcium-free HBSS and imaged under a LSCM, Leica TCS-SP5). The fluorescence intensity (F) was normalized to the baseline fluorescence value F_0_ (F/F_0_) and expressed as mitochondrial calcium concentration ([Ca^2+^]_m_). We measured F_max_ and F_min_, as previously described. F_max_ was obtained by perfusion with 10-μM ionomycin and 5-mM CaCl_2_; F_min_ was measured by perfusion with 10-mM ethylene glycol-bis (β-aminoethyl ether) -N,N,N′,N′-tetraacetic acid (EGTA) and 20-μM 1,2-bis (2-aminophenoxy) ethane-N,N,N′,N′-tetraacetic acid tetrakis (acetoxymethyl ester) (BAPTA-AM; B1205, Molecular probes) in HBSS. Further, 2-aminoethoxydiphenyl borate (2-APB; ab120124, Abcam), TG (T9033, Sigma-Aldrich), and adenosine triphosphate (ATP; A1852, Sigma-Aldrich) were added to the external solution at a proper final concentration. The fluorescence intensity of Rhod2-AM was measured using LSCM. The fluorescence intensity was converted to [Ca^2+^] using the following formula: [Ca^2+^]_m_ = *K*_d_ × (F − F_min_) / (F_max_ − F), where *K*_d_ is the equilibrium dissociation constant of Rhod2 for Ca^2+^, which was 570 nM.

#### Immunofluorescence

Cardiomyocytes were stained with MitoTracker Deep Red FM (500 nM; M22426, Invitrogen) and fixed in 4% paraformaldehyde (P0099, Beyotime Institute of Biotechnology) at room temperature for 10 minutes. They were then permeabilized with 0.1% Triton 100-X (P0096, Beyotime Institute of Biotechnology) at room temperature for 30 minutes. Cells were washed with phosphate-buffered saline (PBS) three times and blocked with blocking buffer (P0102, Beyotime Institute of Biotechnology) for immunostaining at 37°C for 30 minutes. Samples were incubated with anti-MAP1LC3B antibody (1:100) or anti-ERP72 antibody (ab155800, Abcam; 1:100) at 4°C overnight and then washed in PBS twice before staining with the secondary antibody (31561, Invitrogen; 1:500) at 37°C for 2 hours. Co-localization of fluorescence was measured at 100–400 Hz under the LSCM. Samples without primary antibodies were used as negative controls. Images were analyzed using LAS X software (Leica) and Image-Pro Plus 5.0 (Media Cybernetics). Co-localization represented in Pearson’s correlation coefficient was measured using automatic thresholding, as previously described.^55^

#### Measurement of mitochondrial bioenergetics and FAO metabolism

The cellular OCR and extracellular acidification rate (ECAR) were measured using a Seahorse XF96 extracellular flux analyzer (Seahorse Bioscience, North Billerica, USA). Briefly, cells with/without HH exposure or transfected with LVVs-*Pacs2* were plated in XF96-well microplates (6000 cells/well, Seahorse Bioscience). After reaching the proper cell density, the cells were incubated with an XF assay medium without CO_2_ at 37°C for 1 hour. For OCR measurement, cells were then serially exposed to 1-μM oligomycin (mitochondrial/ATP synthase inhibitor), 2-μM trifluoromethoxy carbonyl cyanide phenylhydrazone (FCCP, a mitochondrial uncoupler), and 0.5-μM rotenone/antimycin A (respiratory chain inhibitor), provided in the XF Cell Mito Stress Test Kit (Seahorse Bioscience). Three measurements were performed for each cycle (4-minute mixing, followed by 3-minute detection). The data on basal respiration, maximal respiration, proton respiration, and coupled respiration were collected using Seahorse XF Extracellular Flux analyzer software following the manufacturer’s protocol. To measure the FAO, cells were cultured and replaced by an FAO assay medium containing palmitate-BSA according to the manufacturer’s instructions. Other conditions were consistent with the normal OCR measurement. OCR or ECAR experiments were conducted at 37 °C with adjusted pH of 7.4. Following an XF assay, the number of cells was determined and used to normalize OCR and ECAR.

#### Measurement of mitophagy levels using the mitochondria-targeted Keima reporter

We used the mtKeima reporter to measure the mitophagy levels. Cardiomyocytes were transfected with mitochondria-targeted monomeric Keima-Red-hyg (mtKeima; AM-V0251HM, Medical and Biological Laboratories Co., Ltd.), which contained the hygromycin B-resistance gene. Hygromycin B infection was used to screen and obtain cardiomyocytes stably expressing mtKeima; cardiomyocytes were seeded on glass-bottom dishes and observed under an LSCM to evaluate mitophagy levels. The wavelengths of excitation and emission filters used were as follows: cytoplasmic Keima: 488 nm, 650–760 nm, and lysosomal Keima: 561 nm, 570–630 nm. Images were analyzed using ImageJ software. Briefly, the cardiomyocytes and mtKeima were segmented, and the areas of cytoplasmic and lysosomal mtKeima were determined. The mitophagy index was calculated as the ratio of the total area of lysosomal mitochondria to the total area of cytoplasmic mitochondria per well.

#### Transmission electron microscopy

The right myocardium or H9C2 cardiomyocytes were fixed in 2.5% glutaraldehyde for 2 hours and immersed in 1% osmic acid for 2 hours at 4°C. The fixed samples were then washed in PBS and dehydrated in a graded series of ethanol. Subsequently, the samples were embedded in Epon 812 (SPI Supplies, West Chester, PA, USA) and placed in a model for polymerization. After the semi-thin section was used for positioning, the ultrathin section was made and collected for microstructure analysis, followed by counterstaining with 3% uranyl acetate and 2.7% lead citrate. Subsequently, we observed the sections using an HT7800 TEM (HITACHI, Tokyo, Japan) operating at 100 kV.

#### LC-MS metabolomics analysis

We weighed 60 mg of sample and added 20 μL of 2-chloro-l-phenylalanine (0.3 mg/mL, dissolved in methanol) and 0.6 mL of mixed solution (methanol/water = 7/3 [v:v]) into the 1.5-mL EP tube. The samples were homogenized for 2 minutes and then extracted for 30 minutes by sonication. They were then placed at −20°C for 20 minutes and centrifuged at 13000 g for 15 minutes (4°C). LC-HRMS was performed on a Waters UPLC I-class system equipped with a binary solvent delivery manager and a sample manager, coupled with a Waters VION IMS Q-TOF Mass Spectrometer equipped with an electrospray interface (Waters Corporation, Milford, USA). The injection volume was 3.00 μL, and the column temperature was set at 45°C. The mass spectrometric data were collected using a Waters VION IMS Q-TOF Mass Spectrometer equipped with an electrospray ionization source operating in either positive or negative ion mode. The source and desolvation temperatures were set at 120°C and 500°C, respectively, with a desolvation gas flow of 900 L/h. Centroid data was collected from 50 to 1000 m/z with a scan time of 0.1 s and an interscan delay of 0.02 s over a 13-minute analysis duration.

#### iTRAQ proteomics analysis/nanoUHPLC-MS/MS analysis

Proteomics analyses were performed by Sinotech Genomics Inc. (Shanghai, China) according to the standard procedure and raw data were submitted to the integrated proteome resources (iProX) database (Project ID: IPX0005958000). Briefly, lysis buffer (1% SDS, 8-M urea, 1x Protease Inhibitor Cocktail [Roche Ltd. Basel, Switzerland]) was added to the samples and vibrated and milled for 400 s thrice. The samples were then lysed on ice for 30 minutes and centrifuged at 15000 rpm for 15 minutes at 4°C. The protein concentration of the supernatant was determined using the BCA protein assay; we then transferred 100 μg of protein/condition into a new Eppendorf tube and adjusted the final volume to 100 μL with 8-M urea. We added 2 μL of 0.5-M TCEP and incubated the sample at 37°C for 1 hour; subsequently, 4 μL of 1-M iodoacetamide was added to the sample, and the incubation lasted 40 minutes, protected from light at room temperature. Five volumes of −20°C pre-chilled acetone were then added to precipitate the proteins overnight at −20°C. The precipitates were washed by 1-mL pre-chilled 90% aqueous acetone solution twice and then re-dissolved in 100-μL 100-mM TEAB. Sequence grade modified trypsin (Promega, Madison, WI) was added at the ratio of 1:50 (enzyme: protein, weight: weight) to digest the proteins at 37°C overnight. The peptide mixture was desalted by C18 ZipTip, quantified by Pierce™ Quantitative Colorimetric Peptide Assay (23275), and lyophilized by SpeedVac.

The resultant peptide mixture was labeled with iTRAQ 8Plex labelling kit (Sciex) following the manufacturer’s instructions. The labeled peptide samples were then pooled and lyophilized in a vacuum concentrator. The peptide mixture was re-dissolved in the buffer A (20-mM ammonium formate in water, pH 10.0, adjusted with ammonium hydroxide) and fractionated by high pH separation using Ultimate 3000 system (ThermoFisher Scientific, MA, USA) connected to a reverse-phase column (XBridge C18 column, Waters Corporation, MA, USA). High pH separation was performed using a linear gradient, starting from 5% B to 45% B in 40 minutes (B: 20-mM ammonium formate in 80% ACN, pH 10.0, adjusted with ammonium hydroxide). The peptides were re-dissolved in 5% ACN aqueous solution containing 0.5% formic acid and analyzed by on-line nanospray LC-MS/MS on Q Exactive™ HF-X coupled to EASY-nLC 1200 system (Thermo Fisher Scientific, MA, USA). The column flow rate was maintained at 250 nL/min. The electrospray voltage of 2 kV versus the inlet of the mass spectrometer was used.

#### Bioinformatics data analysis

The UPLC–Q-TOF/MS raw data were analyzed using progenesis QI (Waters CorporationMilford, USA) software. The parameters used were retention time (RT) range 0.5–14.0 minutes, mass range 50–1000 Da, and mass tolerance 0.01 Da. Isotopic peaks were excluded from the analysis, noise elimination level was set at 10.00, minimum intensity was set to 15% of base peak intensity, and RT tolerance was set at 0.01 minute. The excel file was obtained with three-dimensional data sets including m/z, peak RT, and peak intensities; RT–m/z pairs were used as the identifier for each ion. The resulting matrix was further reduced by removing any peaks with missing values (ion intensity = 0) in > 60% of samples. The internal standard was used for data quality control (reproducibility). The positive and negative data were combined to yield a combined data set imported into SIMCA-P + 14.0 software package (Umetrics, Umeå, Sweden). Principle component analysis and (orthogonal) partial least-squares-discriminant analysis ([O] PLS-DA) were performed to visualize the metabolic alterations among the experimental groups, after mean centering and unit variance scaling. Tandem mass spectra were processed by PEAKS Studio version X (Bioinformatics Solutions Inc., Waterloo, Canada). Differentially expressed proteins were filtered if they contained ≥ 1 unique peptide with P ≤ 0.05 and fold change ≥ 1.2. The pathway analysis was performed using GO and the KEGG database.

### Quantification and statistical analysis

All statistical analyses were performed with SPSS 20.0 software (Inc., USA). The measurement variables were presented as mean ± standard deviation (SD) in minimum triplicates. Statistical significance was determined using Student’s *t*-test between two groups and corrected for multiple comparisons (least-significant difference) for more than two groups. Mann–Whiney U test or nonparametric analysis of variance (Kruskal–Wallis) followed by Dunn’s multiple comparison post-hoc test was used when one or more datasets showed non-normal distribution. Imaging experiments and animal tests were assessed in a blinded fashion. Sample sizes were similar between experimental groups and replicates of experiments. The number of biological replicates and observations are described in the figure legends. Statistical significance was considered at *P* < 0.05, with ∗*P* < 0.05, ∗∗*P* < 0.01. For graphs, all data were analyzed using GraphPad Prism software (version 8.4.0; GraphPad Software Inc, San Diego, CA).

## Data Availability

• Proteomics and metabolomics analysis data have been provided in the Tables S1 and S2. The raw data of proteomics were submitted to the integrated proteome resources (iProX) database (Project ID: IPX0005958000). • This paper does not report original code. • All data supports the main and supplemental figures are either available online or available from the corresponding authors upon reasonable request.

## Supplemental information

Document S1. Figures S1–S6 and Data S1

Table S1. The fold changes of differential expression proteomics proteins in the right myocardium of hypobaric hypoxia exposed mice compared with the control mice, related to Figure 1

Table S2. The fold changes of main metabolites in the right myocardium of hypobaric hypoxia exposed mice compared with the control mice, related to Figure 1
