# Supplementary material for: Mitochondria-associated membrane protein PACS2 maintains right cardiac function in hypobaric hypoxia
Source: iScience. 2023 Mar 5;26(4):106328. doi: 10.1016/j.isci.2023.106328 (PMC10034453; doi:10.1016/j.isci.2023.106328)

iScience, Volume *26*

Supplemental information

Mitochondria-associated membrane protein PACS2 maintains right cardiac function in hypobaric hypoxia

Jie Yang, Mengjia Sun, Renzheng Chen, Xiaowei Ye, Boji Wu, Zhen Liu, Jihang Zhang, Xubin Gao, Ran Cheng, Chunyan He, Jingyu He, Xuhong Wang, and Lan Huang

Supplemental Information

Figure S1 related to Figure 2
Figure S2 related to Figure 2
Figure S3 related to Figure 5
Figure S4 related to Figure 6
Figure S5 related to Figure 8
Figure S6 related to Figure 8
Table S1 related to Figure 1
Table S2 related to Figure 1

Figure S1

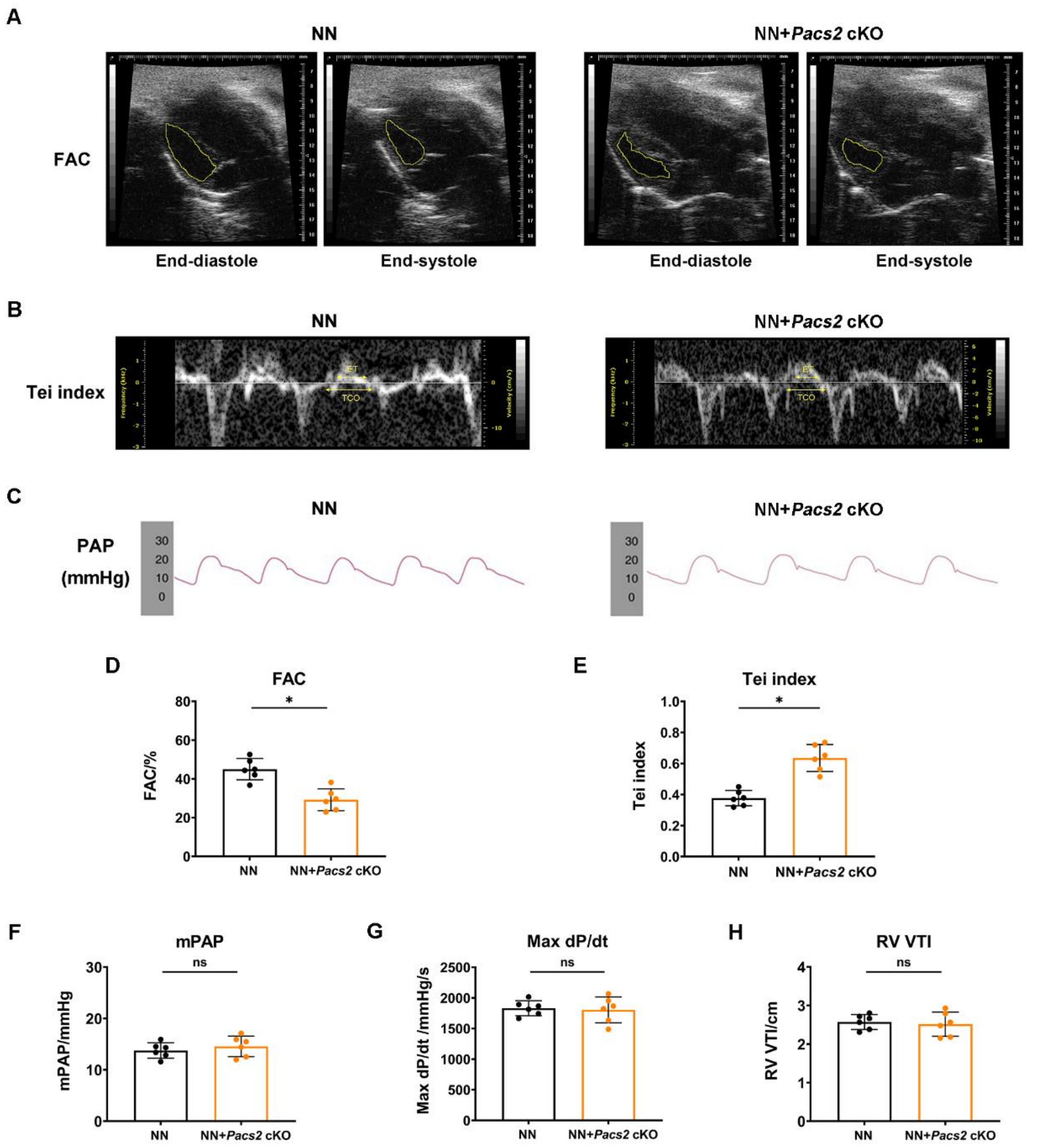

Figure S1. ***Pacs2*** **ablations impaired right cardiac function under normobaric normoxia condition, Related to Figure 2**

(A) FAC measurement of the RV in control group (FAC=40.81%) and *Pacs2* cKO group (FAC=23.47%) in NN condition. Representative images acquired at end-diastole (left) and end-systole (right).

(B) Tei index was measured in control group (Tei index=0.40) and *Pacs2* cKO group (Tei index=0.56) by tissue Doppler imaging.

(C) mPAP measured by RHC of control (mPAP=14.58 mmHg) and *Pacs2* cKO group (mPAP= 15.19 mmHg).

(D-H) Statistics analysis revealed maintained FAC (D) and Tei index (E), but an increased mPAP (F), max dP/dt (G), RV VTI (H) in *Pacs2* cKO mice compared to littermate controls.

Cardiac function indexes were obtained from 6 mice per group, data was showed by mean ± SD, *$P < 0.05$, **$P < 0.01$. NN, normobaric normoxia; RV, right ventricular; HE, hematoxylin-eosin; WGA, wheat germ agglutinin; CSA, cross-sectional area; BNP, brain natriuretic peptide; TnI, troponin I; CK-MB, creatine kinase MB; RHC, right cardiac catheterization; FAC, fractional area change, mPAP, mean pulmonary artery pressure; max dP/dt, maximum positive time derivative of left ventricular pressure; VTI, velocity time integral; ET, ejection time; TCO, tricuspid closure opening time.

Figure S2

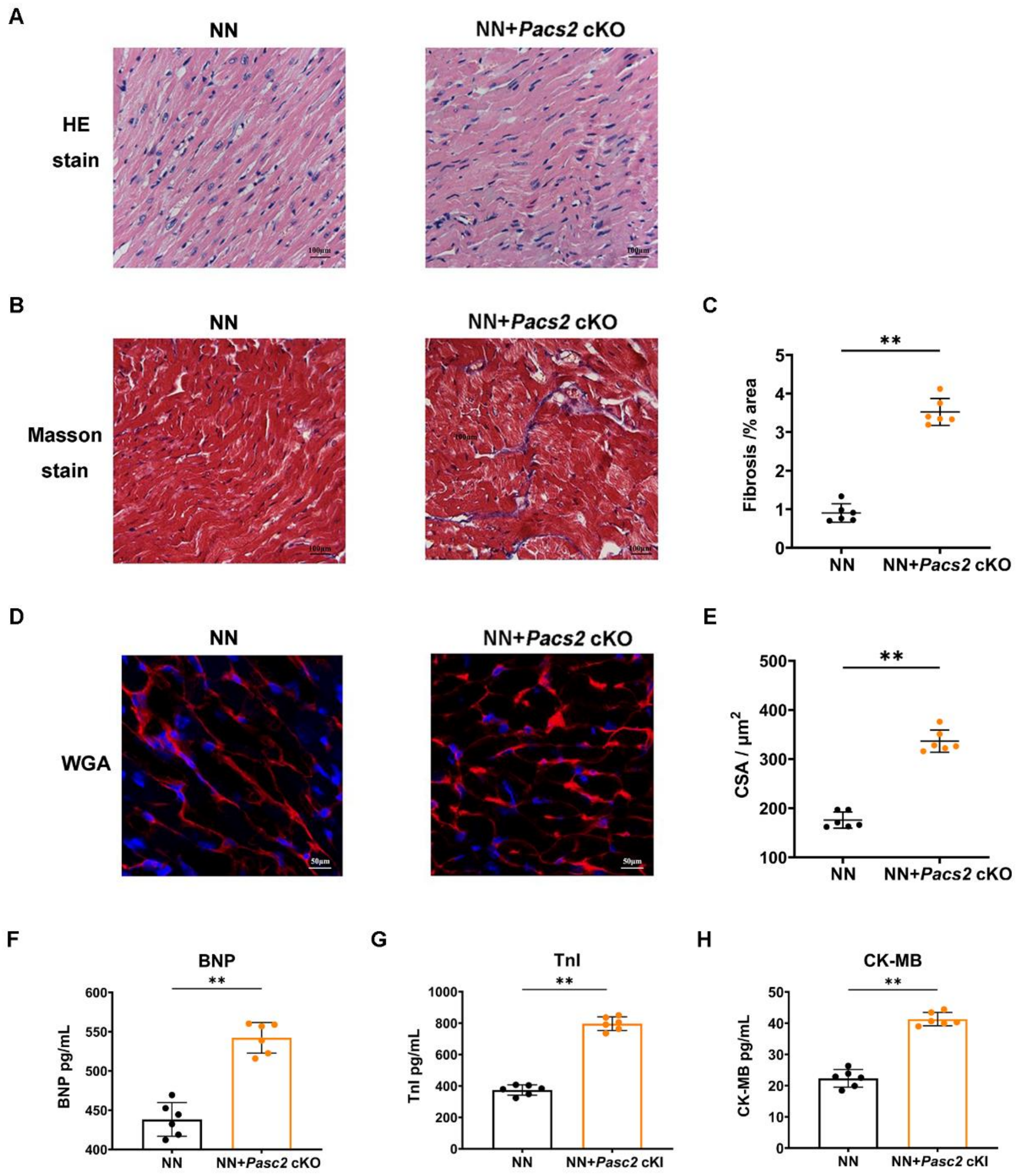

Figure S2. *Pacs2* ablations impaired right cardiac structure under normobaric normoxia condition, Related to Figure 2

(A-B) HE staining (A) and Masson's trichrome staining (B) photographs of RV myocardium. Scale bar, 100 μm.

(C) Quantification of fibrotic area revealed less myocardial fibrosis area (blue) in *Pacs2* cKO mice.

(D) Representative images of RV myocardium stained with WGA (red) to delineate sarcolemma and DAPI (blue). Scale bar, 50 μm.

(E) Bar graphs revealed the relative cardiomyocytes size.

(F-H) Statistics of plasma concentrations of BNP (F), TnI (G) and CK-MB (H) of *Pacs2* cKO mice and their littermate controls in NN condition.

Right myocardium and plasma samples were obtained from 6 mice per group, data was showed

by mean ± SD, *P < 0.05, **P < 0.01. NN, normobaric normoxia; RV, right ventricular; HE, hematoxylin-eosin; WGA, wheat germ agglutinin; CSA, cross-sectional area; BNP, brain natriuretic peptide; TnI, troponin I; CK-MB, creatine kinase MB; RHC, right cardiac catheterization.

Figure S3

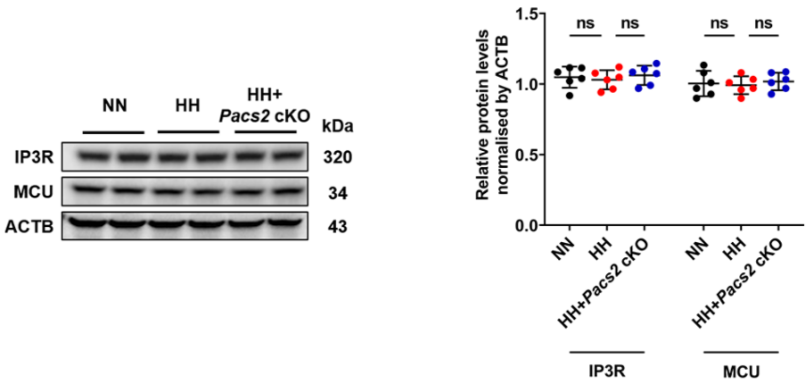

Figure S3. Effects of hypobaric hypoxia and conditional PACS2 knockout on the expression of calcium transport proteins, Related to Figure 5

Representative Western blot and quantitative analysis of IP3R and MCU expression in the NN, HH and HH+*Pacs2* cKO mice heart.

Right myocardium samples were obtained from 6 mice per group, data was showed by mean ± SD. NN, normobaric normoxia; HH, hypobaric hypoxia; IP3R, inositol-1,4,5-triphosphate receptor; MCU, mitochondrial Calcium Uniporter.

Figure S4

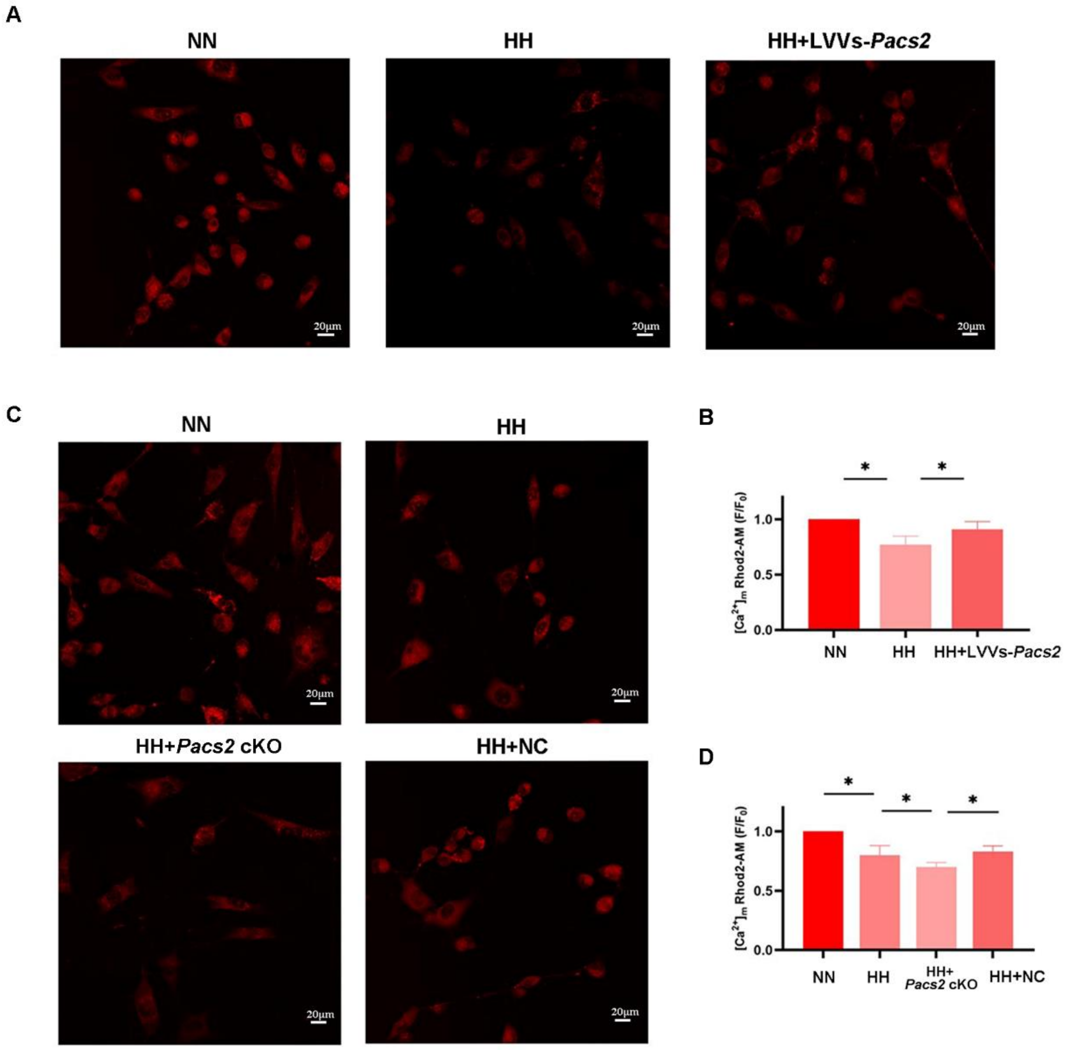

Figure S4. Lentiviral vectors overexpression of Pacs2 increases free mitochondria calcium levels during hypobaric hypoxia exposure, Related to Figure 6

(A) Cells were incubated with the fluorescent calcium indicator, Rhod-2. Calcium concentrations in the mitochondria were observed by LSCM. Scale bar: 20 μm.

(B) Quantitation analysis revealed that LVVs overexpression of *Pacs2* increased free mitochondria calcium levels during hypobaric hypoxia exposure.

(C) Cells were incubated with the fluorescent calcium indicator, Rhod-2. Calcium concentrations in the mitochondria were observed by LSCM. Scale bar: 20 μm.

(D) Quantitation analysis revealed *Pacs2* cKO decreased free mitochondria calcium levels during HH exposure.

Cells were isolated from 6 mice per group, data was showed by mean ± SD, $*P < 0.05$, $**P < 0.01$. LVVs, lentiviral vectors; HH, hypobaric hypoxia; LSCM, laser scanning confocal microscopy.

Figure S5

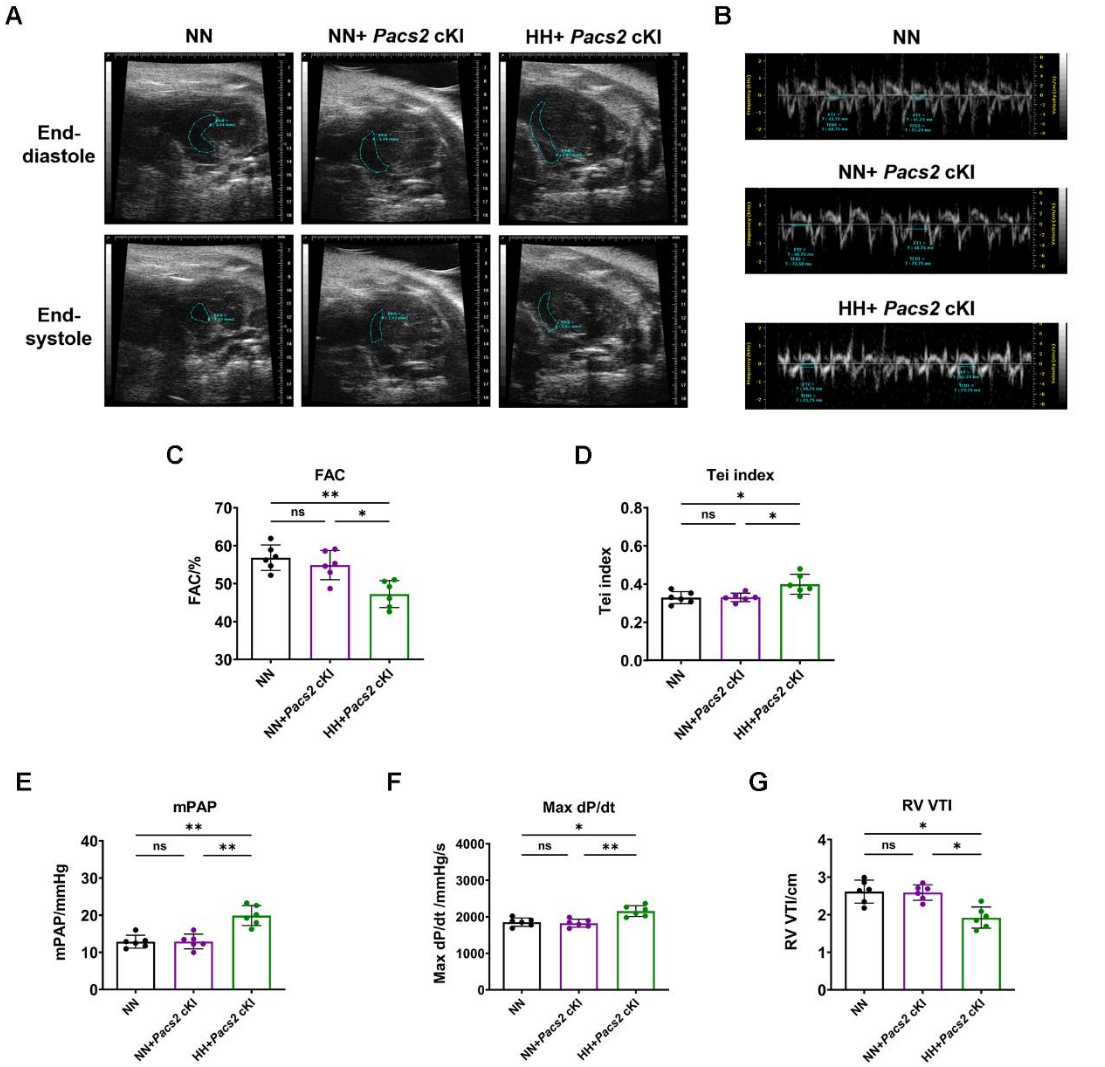

Figure S5. The right cardiac function of *Pacs2* cKI mice under normobaric normoxia and hypobaric hypoxia conditions, Related to Figure 8

(A) FAC measurement of the RV in NN group (FAC=61.98%), NN+*Pacs2* cKI group (FAC=53.92%) in HH+*Pacs2* cKI (FAC=50.61%) group. Representative images acquired at end-diastole (up) and end-systole (down).

(B) Tei index was measured in NN group (Tei index=0.32), NN+*Pacs2* cKI group (Tei index=0.33) in HH+*Pacs2* cKI (Tei index=0.39) group by tissue Doppler imaging.

(C-G) Statistics analysis of the right cardiac function index including FAC (C) and Tei index (D), mPAP (E), max dP/dt (F) and RV VTI (G) among the three groups.

Cardiac function indexes were obtained from 6 mice per group, data was showed by mean ± SD, *P < 0.05, **P < 0.01. NN, normobaric normoxia; HH, hypobaric hypoxia; RV, right ventricular; RHC, right cardiac catheterization; FAC, fractional area change, mPAP, mean pulmonary artery pressure; max dP/dt, maximum positive time derivative of left ventricular pressure; VTI, velocity time integral; ET, ejection time; TCO, tricuspid closure opening time.

Figure S6

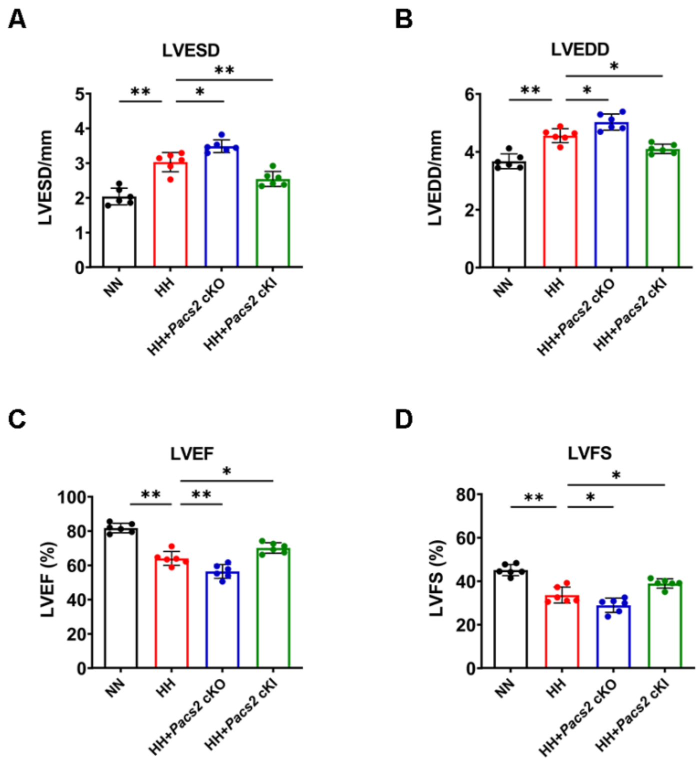

Figure S6. Effects of hypobaric hypoxia and conditional PACS2 genetic manipulation on the left cardiac function in mice, Related to Figure 8

(A-D) Statistics analysis of the left cardiac function index including LVESD (A), LVEDD (B), LVEF (C) and LVFS (D) in the different groups.

Cardiac function indexes were obtained from 6 mice per group, data was showed by mean ± SD, **P* < 0.05. NN, normobaric normoxia; HH, hypobaric hypoxia; LVEDD, left ventricular end-diastolic diameter; LVESD, left ventricular end-systolic diameter; LVEF, left ventricular ejection fraction; LVFS, left ventricular fractional shortening.

Supplemental Tables

Table S1. The fold changes of differential expression proteomics proteins in the right myocardium of hypobaric hypoxia exposed mice compared with the control mice, Related to Figure 1

Table S2. The fold changes of main metabolites in the right myocardium of hypobaric hypoxia exposed mice compared with the control mice, Related to Figure 1

Data S1, Full unedited gels related to Figure 3A, Figure 4A and Supplemental Figure 3

Full unedited gels related to Figure 3A

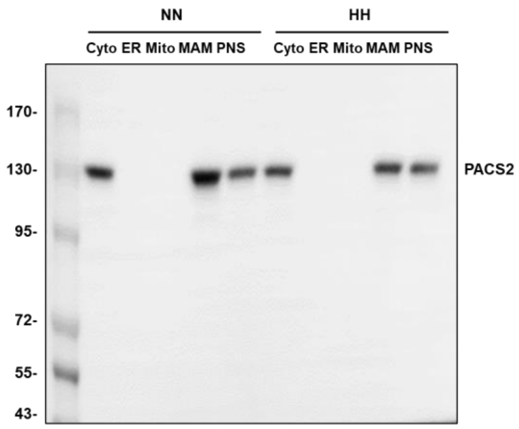

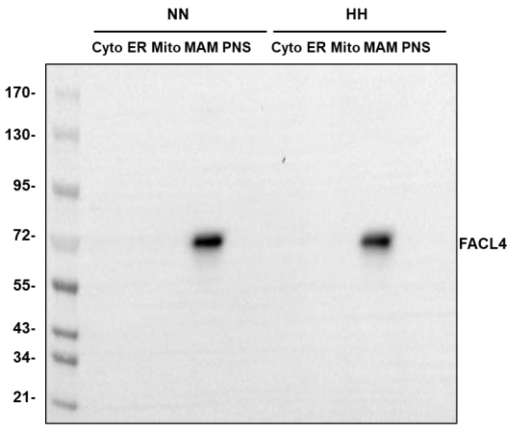

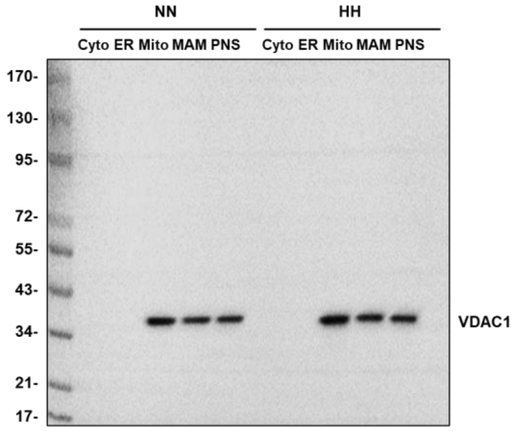

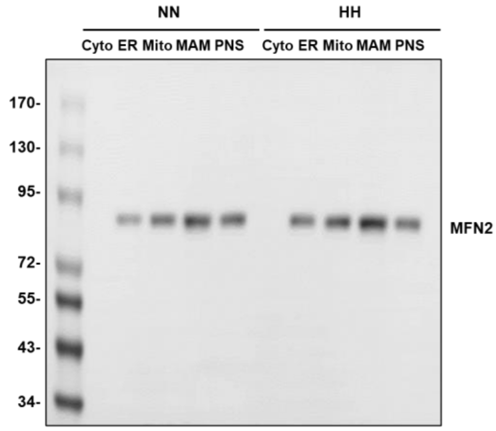

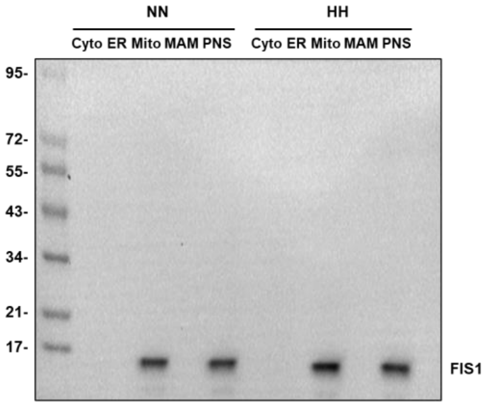

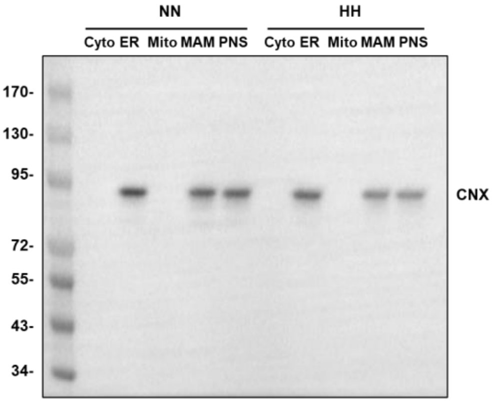

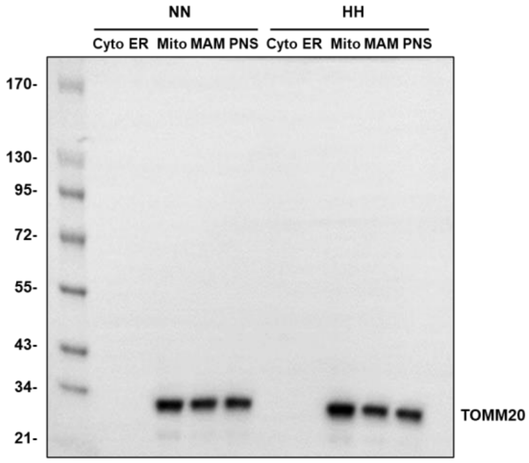

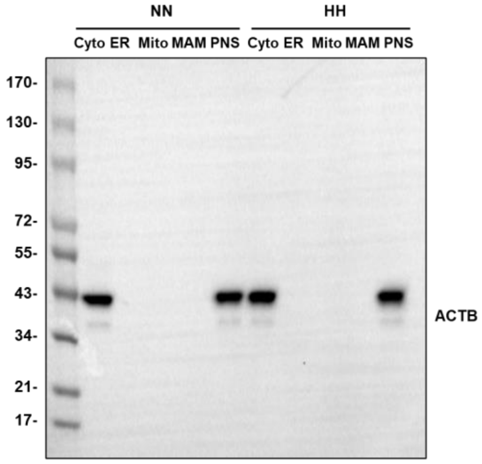

Full unedited gels related to Figure 4A

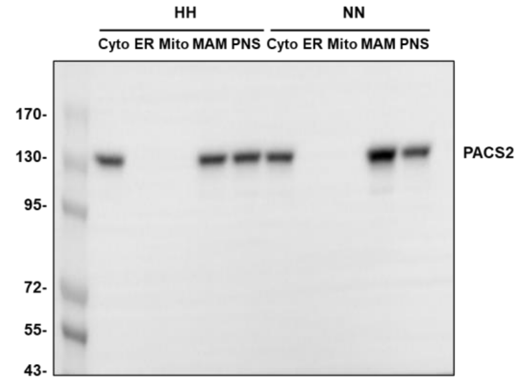

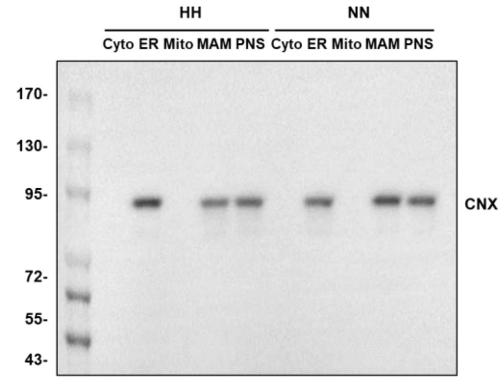

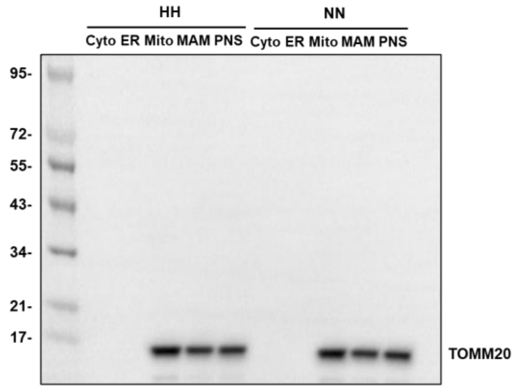

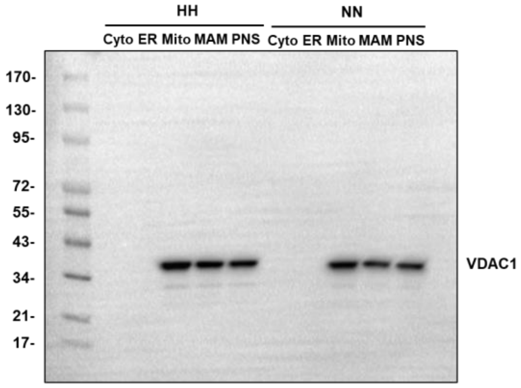

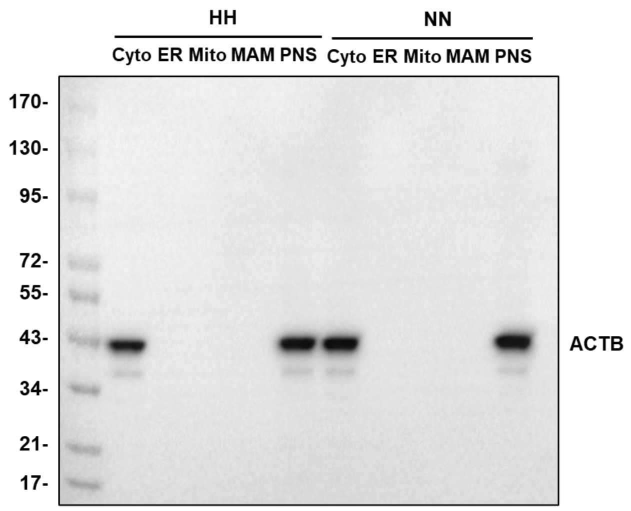

Full unedited gels related to Supplemental Figure 3

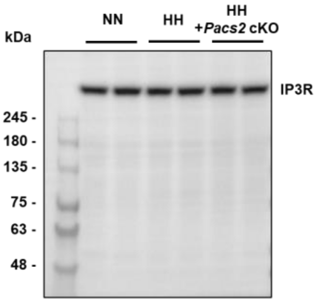

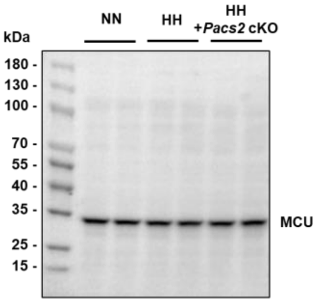

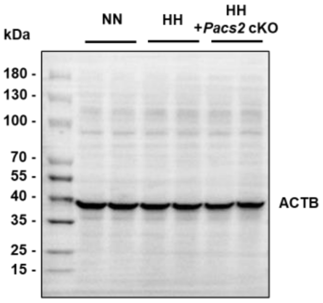

Supplement: Document S1. Figures S1–S6 and Data S1 [file mmc1.pdf]
